# Supplementary figures and images for: Shared decision-making for people living with dementia in extended care settings: a systematic review
Source: BMJ Open. 2018 Jun 9;8(6):e018977. doi: 10.1136/bmjopen-2017-018977 (PMC6009462; doi:10.1136/bmjopen-2017-018977)

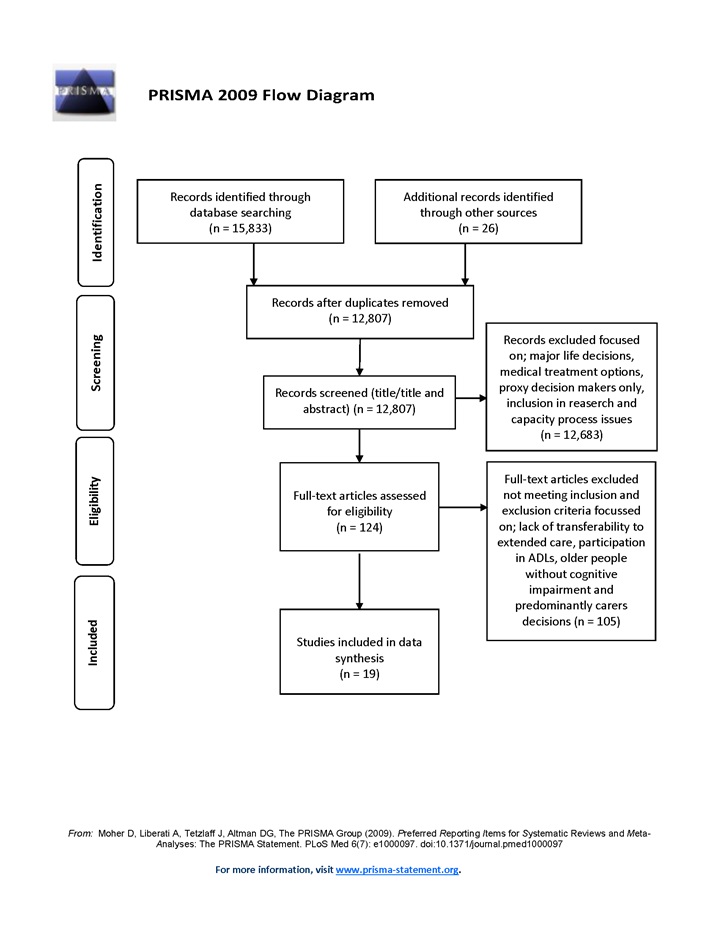

Supplement: Supplementary data [file bmjopen-2017-018977supp001.jpg]
