# Supplementary material for: Shared decision-making for people living with dementia in extended care settings: a systematic review
Source: BMJ Open. 2018 Jun 9;8(6):e018977. doi: 10.1136/bmjopen-2017-018977 (PMC6009462; doi:10.1136/bmjopen-2017-018977)
Supplement: Author's manuscript [file bmjopen-2017-018977.draft_revisions.pdf]

BMJ Open

BMJ Open is committed to open peer review. As part of this commitment we make the peer review history of every article we publish publicly available.

When an article is published we post the peer reviewers' comments and the authors' responses online. We also post the versions of the paper that were used during peer review. These are the versions that the peer review comments apply to.

The versions of the paper that follow are the versions that were submitted during the peer review process. They are not the versions of record or the final published versions. They should not be cited or distributed as the published version of this manuscript.

BMJ Open is an open access journal and the full, final, typeset and author-corrected version of record of the manuscript is available on our site with no access controls, subscription charges or pay-per-view fees (http://bmjopen.bmj.com).

If you have any questions on BMJ Open's open peer review process please email info.bmjopen@bmj.com

BMJ Open

SHARED DECISION-MAKING FOR PEOPLE LIVING WITH COGNITIVE IMPAIRMENT IN EXTENDED CARE SETTINGS: A SYSTEMATIC REVIEW

Journal:	*BMJ Open*
Manuscript ID	bmjopen-2017-018977
Article Type:	Research
Date Submitted by the Author:	03-Aug-2017

Complete List of Authors:	Daly, Rachel; University of Hertfordshire, CRIPACC<br>Bunn, Frances; University of Herfordshire, Department of Health and Human Sciences<br>Goodman, Claire; University of Hertfordshire, Centre for Research in Primary and Community Care

**Primary Subject Heading**:	Patient-centred medicine
Secondary Subject Heading:	Mental health, Evidence based practice, Communication, Geriatric medicine
Keywords:	Dementia < NEUROLOGY, Delirium & cognitive disorders < PSYCHIATRY, shared decision-making, Care Homes, Extended care, Everyday care

SCHOLARONE™
Manuscripts

Title: SHARED DECISION-MAKING FOR PEOPLE LIVING WITH COGNITIVE IMPAIRMENT IN EXTENDED CARE SETTINGS: A SYSTEMATIC REVIEW.

Authors:

Rachel Daly, Queens Nurse, NIHR RDCP programme CLAHRC East of England doctoral student r.daly2@herts.ac.uk

Centre for Research in Primary and Community Care (CRIPACC), University of Hertfordshire, College Lane, Hatfield, Herts, England, AL10 9AB

Professor Frances Bunn, Professor in Health and Complex Conditions f.bunn@herts.ac.uk

Centre for Research in Primary and Community Care (CRIPACC), University of Hertfordshire, College Lane, Hatfield, Herts, England, AL10 9AB

Professor Claire Goodman, Professor Health Services Research c.goodman@herts.ac.uk

Centre for Research in Primary and Community Care (CRIPACC), University of Hertfordshire, College Lane, Hatfield, Herts, England, AL10 9AB

Corresponding author Rachel Daly – r.daly2@herts.ac.uk Tel: +44 7967 761298

Keywords: Cognitive Impairment, Dementia, Shared Decision-Making, Care Homes, Everyday Care, Extended Care, Day-to-Day Care, Residential Care

Tables – 3

References – 57

Supplementary files – Figure 1 (Prisma flow chart)

For peer review only

ABSTRACT

Objectives - Shared decision-making is recognized as an important element of person-centered dementia care. The aim of this review was to explore how people living with dementia and cognitive impairment can be included in day-to-day decisions about their health and care in extended care settings

Design – A systematic review including primary research relating to shared decision-making, with cognitively impaired adults in (or transferrable to) extended care settings. Databases searched were CINAHL, PubMed, the Cochrane Library, NICE Evidence, OpenGrey, Autism Data, Google Scholar, Scopus and Medicines Complete for English language studies published in the last 20 years.

Results. Of the 19 included studies 7 involved people with living dementia in extended care settings. Evidence suggests that people living with cognitive impairment often have the desire and ability to participate in decision-making about their everyday care, although this is regularly underestimated by their staff and family care partners. Shared decision-making has the potential to improve quality of life for both the person living with dementia and those who support them. How resources to support shared decision-making are implemented in extended care settings, is less well understood.

Conclusions – Evidence suggests that people living with cognitive impairment value opportunities to be involved in everyday decision-making about their care. How these opportunities are created, understood, supported and sustained in extended care settings remains to be determined.

Strengths and limitations;

- A systematic search for, and review of, cross-disciplinary literature relating to shared decision-making for people living with cognitive impairment in extended care settings.
- Presentation of the limited shared decision-making resources, tools and measures applicable to people living with cognitive impairment in extended care settings to inform future research and multi-disciplinary practice.
- The quality assessment tool for qualitative studies was changed from the protocol.

Word Count – 4,391

For peer review only - http://bmjopen.bmj.com/site/about/guidelines.xhtml

SHARED DECISION-MAKING FOR PEOPLE LIVING WITH DEMENTIA IN EXTENDED CARE SETTINGS: A SYSTEMATIC REVIEW.

Background

For people living with dementia shared decision-making is increasingly considered crucial in health and care practice [1-4] and as an essential aspect of person centred care [5]. The fundamental premise of which is that the individual's priorities, interests, abilities and character should inform decision-making [6].

A dementia diagnosis does not automatically render someone incapable of making a decision [7, 8] but for a decision to truly be shared, mutuality must be established [9]. Legislative changes in the last decade have strengthened the rights of people living with dementia to participate in decisions about their care [10, 11]. The United Nations Convention on the Rights of People with Disabilities (CRPD), states that "persons with disabilities enjoy legal capacity on an equal basis with others in all aspects of life" [12]. However, evidence on *how* care decisions are currently made within various legislative frameworks, for and with, people living with dementia, is patchy and often focuses on decisions relating to life events [13, 14]. In England and Wales the Mental Capacity Act (2005) provides a legislative framework to protect and empower people to participate in decisions about their life and care unless there is evidence to the contrary [15]. Evidence suggests however, that staff in extended care often make day-to-day care decisions on behalf of people living with dementia [17, 18]. There are situations where people living with dementia in extended care settings decline to participate in decision-making and prefer to relinquish control and delegate to a care partner or worker. Reasons identified include; anxiety about ability to participate and reluctance to accept deceasing abilities (reported as self-protection) [see e.g. 19, 20, 21].

Two reviews have focused on shared decision-making with people living at home with dementia [21, 22]. They offer useful insight into degrees of involvement in the decision-making process, what influences that involvement and how people participate [21, 22].In a review of 36 studies of people living with dementia and their carers Miller [21] identified assorted patterns of decision-making ranging from 'being free to make a choice' through 'supported autonomy' to 'being reliant upon carers'. Larsson's review of 24 studies on

For peer review only - http://bmjopen.bmj.com/site/about/guidelines.xhtml

community decisions relating to access to care services developed three themes to capture how people living with dementia experience involvement in care decisions; *excluded, prior preferences taken into account,* and *current preferences respected* [23]. These reviews usefully bring shared decision-making with people living with dementia into focus but largely excluded people living in extended care settings, who are often frailer and further along the dementia trajectory. They also highlight that decisions for people living with dementia at home invariably involved a care partner/family member. People who are unavailable for many of the day-to-day decisions undertaken in extended care due to time and geographical constraints.

There is increasing recognition that everyday decisions are ubiquitous, thereby potentially making these daily decisions more significant in everyday quality of life than the more noteworthy issues such as treatment decisions or relocation [see e.g. 24, 25, 26]. Building on previous work, this paper considers the evidence on *how* day-to-day decisions are understood and negotiated between people with a cognitive impairment and their (staff and family) carers in extended care settings. The review objectives are presented in *Box 1.*

BOX 1 – Review Objectives

- Explore how shared decision making is understood and/or characterised for people living with dementia and their (staff and family) carers
- Explore the role of (staff and family) carers of people living with dementia in shared decision making care dyads
- Analyse identified risks and benefits associated with shared decision making for people with cognitive impairment
- Ascertain empirical evidence for the effectiveness of available shared decision making resources for people living with dementia
- Seek to understand the barriers and facilitators to effective shared decision making for people living with dementia and their (staff and family) carers
- Explore the extent to which shared decision making has been researched in extended care settings
- Identify implications for shared decision making in dementia care practice, policy and future research

Methods

A detailed account to the review methods are published elsewhere [27] PROSPERO registration number CRD42016035919. The review was planned and is reported in accordance with the Preferred Reporting Items for Systematic Reviews and Meta-Analyses (PRISMA) guidelines [28].

For peer review only - http://bmjopen.bmj.com/site/about/guidelines.xhtml

Study selection and inclusion criteria

Inclusion criteria are presented in *Table 1* below. A time limit of 20 years was applied to reflect the rapidly changing literature on involving people living with dementia in their care, published since the seminal works of Tom Kitwood [29-31].

Table 1 - Inclusion Criteria	
**Publication Language**	English
**Publication Dates**	January 1996 - October 2016
**Target Population**	Adults, aged over 18 years, with any type of cognitive impairment for example dementia, learning disabilities, Parkinson's and Huntingdon's disease
**Study Setting**	Community living at home or extended care settings e.g. supported living, or residential care. Studies must be considered to be transferable to people living with a cognitive impairment in an extended care setting (*a priori* criteria applied)
**Study Types**	Primary research regardless of methodology, investigating or measuring shared decision-making. Including, but not limited to, models, tools or interventions.
**Primary Outcomes Sought**	The person living with cognitive impairment demonstrating; involvement in care planning, receiving care congruent with their expressed choices, improved quality of life or wellbeing, and/or behavioural changes (e.g. reduction in distress)
**Additional Outcome Areas of Interest**	Family care partner and/or health or care professional satisfaction, and any documented adverse effects (e.g. falls, weight loss, adverse outcomes related to medication management)

Table 1–study inclusion criteria

Extended care settings are defined as residential settings that provide onsite care. This includes supported living, care villages and extra care housing in addition to more traditional care homes with and without nursing [32]. Studies from other settings, e.g. people's own homes, are included if they meet all other criteria and offer transferable learning for an extended care setting. For a study to be transferrable the person living with a cognitive

For peer review only - http://bmjopen.bmj.com/site/about/guidelines.xhtml

impairment must be in receipt of care in addition to that provided by a family care partner and the resource should be suitable for implementation in extended care settings.

The term 'care partner’ has been adopted to represent a family member or friend who has a caring relationship with the person living with a cognitive impairment. The terms health and/or care professionals, staff or workers are used interchangeably to describe people who are paid to provide health or social care.

Search strategy

The search strategy drew on a range of cross discipline data sources associated with cognitive impairment and dementia care. Electronic databases searched and an example of the search query for PubMed is given in *table 2*. Search terms were adapted as appropriate for other databases.

Table 2 - Search Strategy	
**Electronic databases searched**	CINAHL Plus, PubMed, the Cochrane Library, NICE Evidence, OpenGrey, Autism Data, Google Scholar, Scopus and MedicinesComplete
**MeSH search terms (with subheadings included)**	Cognition Disorders searched (((("dementia"[MeSH Terms] OR "dementia"[All Fields]) OR ("cognitive dysfunction"[MeSH Terms] OR ("cognitive"[All Fields] AND "dysfunction"[All Fields]) OR "cognitive dysfunction"[All Fields] OR ("cognitive"[All Fields] AND "impairment"[All Fields]) OR "cognitive impairment"[All Fields])) OR ("learning disorders"[MeSH Terms] OR ("learning"[All Fields] AND "disorders"[All Fields]) OR "learning disorders"[All Fields] OR ("learning"[All Fields] AND "disability"[All Fields]) OR "learning disability"[All Fields])) OR ("learning disorders"[MeSH Terms] OR ("learning"[All Fields] AND "disorders"[All Fields]) OR "learning disorders"[All Fields] OR ("learning"[All Fields] AND "disorder"[All Fields]) OR "learning disorder"[All Fields])) OR ("autistic disorder"[MeSH Terms] OR ("autistic"[All Fields] AND "disorder"[All Fields]) OR "autistic disorder"[All Fields] OR "autism"[All Fields])) OR ("stroke"[MeSH Terms] OR "stroke"[All Fields])) OR ("brain injuries"[MeSH Terms] OR ("brain"[All Fields] AND "injuries"[All Fields]) OR "brain injuries"[All Fields] OR ("brain"[All Fields] AND "injury"[All Fields]) OR "brain injury"[All Fields])) OR ("neurocognitive disorders"[MeSH Terms] OR ("neurocognitive"[All Fields] AND "disorders"[All Fields]) OR "neurocognitive disorders"[All Fields])) OR ("alzheimer disease"[MeSH Terms] OR ("alzheimer"[All Fields] AND "disease"[All Fields]) OR "alzheimer disease"[All Fields] OR "alzheimer"[All Fields])) AND Shared Decision-making ((((((shared[All Fields] AND ("Decision (Wash D C)"[Journal] OR "decision"[All Fields])) OR ("decision-making"[MeSH Terms] OR ("decision"[All Fields] AND "making"[All Fields]) OR

For peer review only - http://bmjopen.bmj.com/site/about/guidelines.xhtml

	"decision-making"[All Fields])) OR ("community participation"[MeSH Terms] OR ("community"[All Fields] AND "participation"[All Fields]) OR "community participation"[All Fields] OR ("consumer"[All Fields] AND "participation"[All Fields]) OR "consumer participation"[All Fields])) OR ("patient participation"[MeSH Terms] OR ("patient"[All Fields] AND "participation"[All Fields]) OR "patient participation"[All Fields])) OR (("Decision (Wash D C)"[Journal] OR "decision"[All Fields]) AND support[All Fields])) OR (care[All Fields] AND dyad[All Fields])) OR ("patient education handout"[Publication Type] OR "patient education as topic"[MeSH Terms] OR "patient education"[All Fields]))) NOT (((advance[All Fields] AND ("Decision (Wash D C)"[Journal] OR "decision"[All Fields])) OR ("advance directives"[MeSH Terms] OR ("advance"[All Fields] AND "directives"[All Fields]) OR "advance directives"[All Fields] OR ("advance"[All Fields] AND "directive"[All Fields]) OR "advance directive"[All Fields])) OR (advance[All Fields] AND care[All Fields] AND plan[All Fields]))) OR ((((((("Dementia"[Mesh] OR "Neurocognitive Disorders"[Mesh]) OR "Brain Injuries"[Mesh]) OR "Stroke"[Mesh]) OR "Learning Disorders"[Mesh]) OR "Autistic Disorder"[Mesh]) AND ((((("Decision-making"[Mesh] OR "Decision Support Techniques"[Mesh]) AND "Patient Participation"[Mesh]) OR "Cooperative Behavior"[Mesh]) OR "Physician-Patient Relations"[Mesh]) OR "Patient Education as Topic"[Mesh])) NOT Advance Directives("Advance Directives"[Mesh] OR "Advance Care Planning"[Mesh]))) NOT Paediatrics ("child"[MeSH Terms] OR "child"[All Fields])) NOT ("Child"[Mesh] OR "Disabled Children"[Mesh])
**Alternate free text search terms**	(Cogniti*, Disorder*, Dementia*, Alzheimer*, Neurocogniti* Dis*, Brain Injur*, Autis*, Learning Dis*, Stroke) AND (Shared Decision-making, Deci* Mak*, Patient Participat*, Consumer Participat* , Cooperat*, Decision Support) NOT (Paed* Child*) NOT (Advance Directives, Advance* care planning, Advance* deci*)
**Google Scholar**	The search and screening strategy for Google Scholar was agreed by all 3 authors. Free text search terms mirrored other databases. Results were filtered by relevance. The first 20 pages of results, title and abstract, were screened (20 results per page).

Table 2- Electronic databases searched, and search terms used

Reference lists of relevant primary and review articles were manually searched to detect additional studies and citation search was performed using the 'cited by' option on Google Scholar, and the 'related articles' option in PubMed. Searches were initially undertaken in June 2016 and updated in October 2016. Lateral searches were completed in November 2016.

For peer review only - http://bmjopen.bmj.com/site/about/guidelines.xhtml

Electronic search results were downloaded into EndNote. One author (RD) screened all references and, where necessary, sought support and independent review from a 2nd author (FB or CG).

Quality Assessment and Data Extraction

Quality assessment was undertaken using the validated QualSyst Framework [33-35]. The QualSyst framework provides comprehensive definitions, instructions, and a scoring system for quality scoring of both quantitative and qualitative studies represented as a percentage (with a greater percentage representing higher quality)[35]. An ethical approval question was added as this has been highlighted as an essential element of study quality [36, 37]. Quality assessment was carried out by one reviewer (RD), with 10% checked by a second reviewer (FB/CG).

Data were extracted using a structured form that addressed the review objectives. This included information about the study design, participants and outcomes.

Analysis

Due to heterogeneity, and low numbers, of included quantitative studies, meta-analysis was not appropriate therefore all results are reported in a narrative format.

Studies were compiled and evaluated using theoretical thematic analysis using the research question and review objectives (see *box 1*) as a framework to map the range of data and identify recurrent themes. Whilst this method provides a less detailed description of the studies overall than inductive analysis, it offers greater detail of the identified areas of interest [38]. In this instance exploring the concepts of understanding shared decision-making for people living with cognitive impairment; roles, resources, barriers, facilitators and risks, relevant to extended care settings.

Results

Nineteen publications are included [10, 11, 24, 39-54](see *table 3*) relating to 18 unique studies (including a doctoral dissertation). Four of the papers are linked as they included a

For peer review only - http://bmjopen.bmj.com/site/about/guidelines.xhtml

baseline sample drawn from the same pool of participants [24, 42, 49, 54]. However, the studies addressed different questions and are, therefore, included as individual papers. An overview of the screening and selection process is demonstrated in Figure 1.

(insert) Figure 1 - PRISMA flow chart

Study Quality

Study quality ranged from 65-100% see *table 3*. No studies were excluded as a result of the quality assessment but a high risk of bias was noted in some studies, such as a measure designed by the same people as the intervention [43]. Eight studies did not provide a clear ethics statement [11, 24, 41-43, 49, 51, 54], including the two studies that scored 100% on QualSyst [49, 54].

Characteristics of included studies and participants

All included studies (see *table 3*) were published between 2002 and 2016. The majority (n=14) of studies were published in the last 10 years, suggesting an increasing awareness and interest in shared decision-making for people living with a cognitive impairment that reflects the progression in national and international legislation. All five UK studies were published since the full implementation of the Mental Capacity Act [15].

Most papers (n=15) focus on people living with dementia [10, 11, 24, 40-42, 44-47, 49, 51-54]; twelve are in 'care dyads' (with a care partner) or 'triads' (with a care partner and a health or care worker). Two papers centre on people with a learning disability [39, 48], one on people living with Huntingdon's disease [43] , and one includes some people with cognitive impairment [50]. Seven studies represent some participants living in extended care settings [10, 11, 41, 45, 46, 48, 50, 53], whilst the majority (n=12) relate to people living at home. Shared decision-making in extended care settings was the focus of only two studies [41, 45].

The majority of studies (n=15) employed interviews and/or observations (see *table 3*). Three of the linked studies used structured interviews and compared the views of people living with a cognitive impairment and their care partners [42, 49, 54]. One study, used structured observations of family meetings [50]. Three studies undertook ethnographic observations of

For peer review only - http://bmjopen.bmj.com/site/about/guidelines.xhtml

people living with dementia within a care dyad or triad [10, 40, 41]. Four studies identified person centred care as a theoretical framework [40, 45, 51, 53].

A breakdown of whether studies aimed to understand, evaluate interventions or measure shared decision-making is presented in *table 3* and discussed within the narrative below. Results are presented in cross-cutting themes that explore decision-making participation or involvement for people living with a cognitive impairment. Results are presented to reflect relevance to the review question and objectives rather than frequency that the topic arises in the data [38].

Table 3 - Characteristics of included studies

Author(s) and year	Purpose of study	Country	Methods	Quality score	Setting	Participants	Sample size			
							PLWCI	CP	CW	Total
**Clarke, A. M. (2004)**	To influence SDM	Australia 3	Qualitative Interviews and observations	75%	extended care	PLWD and CW	13		13	26
**Fetherston-haugh, D. (2013)**	To understand SDM		Qualitative Interviews	80%	home	PLWD		6		6
**Milte, C. (2015)**	To understand SDM		Quantitative Observed family meetings	83%	intermediate care	Older people, CPs and HCPs	51	51	2	104
**Tyrrell, J. (2006)**	To measure SDM	France 1	Qualitative Interviews	83%	home	PLWD and care partner	21	21		42
**Span, M. (2016)**	To facilitate SDM	Holland 1	Qualitative Interviews, focus groups, specialist consultation and workshops	75%	home	PLWD, CPs and HCPs				84
**Smebye, K. L. (2012)**	To understand SDM	Norway 1	Mixed methods Interviews and observations	95%	home and extended care	PLWD, CPs and HCPs	10	10	10	30
**Ferm, U. (2010)**	To facilitate SDM	[illegible] den	Mixed methods Interviews	95%	home	PLW HD	5			5

For peer review only - http://bmjopen.bmj.com/site/about/guidelines.xhtml

**Kjellberg, A. (2002)**	To understand		Qualitative Interviews	70%	home, extended and day care	People living with LD	23			23
**Bailey, R. (2011)**	To measure SDM	United Kingdom 5	Quantitative Electronic decision-making tasks and questionnaire	79%	Day services	People living with LD	24			24
**Boyle, G. (2014)**	To measure SDM		Qualitative Creative interaction, observation and interviews	85%	home	PLWD and CPs	5	5		10
**Godwin, B. (2014)**	To facilitate SDM		Mixed methods Consultation	90%	extended care	PLWD	34		42	76
**Murphy, J. (2013)**	To facilitate SDM		Mixed methods Researcher facilitated discussion	65%	home	PLWD and CPs	18	18		18
**Samsi, K. (2013)**	To understand SDM		Qualitative Interviews	90%	home	PLWD and CPs	15	15		30
**Feinberg, L. F. (2002)**	To measure SDM	United States of America 6	Quantitative Interviews	80%	home	PLWD and CPs	51	51		102
**Hirschman, K. B. (2005)**	To understand SDM		Interviews	70%	Home and extended care	PLWD and CPs	48	48		96
**Horton-Deutsch, S. (2007)**	To understand SDM		Mixed methods Interviews	85%	home	PLWD and CPs	20	20		40
**Menne, H. L. (2008)**	To measure SDM		Quantitative Interviews Demographic information inc. MMSE scores Capacity evaluation	100%	home	PLWD and CPs	217	217		434
**Menne, H. L. (2007)**	To measure SDM		Quantitative Secondary data analysis	86%	home	PLWD and CPs	215	215		430
**Whitlatch, C. (2005)**	To measure SDM		Mixed methods Interviews	100%	home	PLWD and CPs	111	111		222

HD – Huntingdon's Disease, SDM – shared decision-making, PLWCI – person living with cognitive impairment, PLWD – person living with dementia, LD – Learning disability CP – care partner, CW –care worker, HCP – health care professional

Table 3 - Characteristics of included studies

For peer review only - http://bmjopen.bmj.com/site/about/guidelines.xhtml

Understanding 'shared decision-making' for people living with cognitive impairment

There was no common understanding of what shared decision-making entails and how it can be operationalised for people living with a cognitive impairment. Only one paper offered a definition of shared decision-making. Defining it as an approach that involves patients in making medical decisions with their clinician [55], is influenced by the type and complexity of the decision being made and the perspectives of care partners as well as the person living with cognitive impairment.

Three ways that people living with dementia understand shared decision-making were identified in a phenomenological study [44]; subtle support versus taking over; hanging on versus letting go; and being central versus being marginalised or excluded. One participant described their negative response to being excluded;

> *"…if someone came in and started telling me how I should run things or do things, I think I would certainly retaliate and not conform to anything they would want to do."* [44 p.147].

A recurrent theme in the qualitative literature reviewed is that for many people living with dementia, it is the participation or 'sharing' in the decision-making process, that is as, (if not more) important than making the decision itself [11, 44, 47, 56]. Despite this Samsi and Manthorpe [52] identified examples of 'substituted decision-making' that essentially excluded the person living with dementia;

> *"Oh I don't ask her what she wants anymore. I know what she'll say anyway - 'anything you like, you decide', so I just do what's best for us both."* [52 p958]

Participation in decision-making

The extent to which a person living with dementia is able to participate in decision-making was a focus of many of the included studies. For the purpose of this paper participation in decision-making is subdivided into the degree or level of participation, ability to participate and desire to participate.

For peer review only - http://bmjopen.bmj.com/site/about/guidelines.xhtml

Levels of participation in decision-making

Five studies addressed levels of participation [10, 11, 47, 48, 52]. Smebye and colleagues used Thompson's five point taxonomy of patient involvement and participation in health care consultations [57]. Thompsons taxonomy ranges from entirely passive 'non-involvement', through cooperative 'shared decision-making', to independent 'autonomous decision-making' [57]. Smebye identified two additional elements through care triad observations and interviews; pseudo-autonomous, (assumptions about a person's decision) and delegated (the person living with dementia expressly delegates their decision-making) when including people living with dementia in care triads [10]. This extended taxonomy is reflected in all five studies exploring the extent of decision-making participation.

Shared-decision-making was considered as the most common decision-making pattern by people living with a cognitive impairment. Horton-Deutsch and colleagues [47] explored self-reported participation in decision-making for 20 people living with dementia and their care partners' using semi-structured interviews and a five point decision making scale centred on a treatment vignette. The scale ranged from (1) made decisions alone in the past with little input from others and continue to do so, to (5) discussed decisions with spouse in the past and continues to do so. Participants reported that although their self-perceived decision-making changed over time from, largely independent historically towards current interdependent (shared) decision-making, the majority (75%) of participants described some level of shared decision-making throughout [47]. Similarly, in a group of 23 people living with varying levels of learning disability, 70% rarely considered themselves entirely independent and relied on shared decision-making in some or all areas of their life [48].

Ability to participate

Seven studies [24, 39-42, 46, 49] explored individuals' ability to participate in decisions often in the context of facilitators or barriers [e.g. 41]. All the studies presenting measures of decision-making participation compared the responses of the person living with a cognitive impairment with their care partners. They highlighted consistent incongruence between responses, with care partners typically believing the person living with dementia to be less involved than they perceived themselves [46, 54].

For peer review only - http://bmjopen.bmj.com/site/about/guidelines.xhtml

Five papers describe and/or evaluate tools that measure aspects of decision-making participation from the perspective of the person living with a cognitive impairment [11, 24, 42, 49, 54]. The linked studies (with an overlapping sample ranging from 51-217 care dyads) develop, use and evaluate the Decision Control Inventory (DCI) and the Decision-Making Involvement Scale (DMI) to assess and compare everyday care choices of both the person living with a cognitive impairment and their care partner [24, 42, 49, 54]. The DMI Scale measures involvement in everyday decisions such as what food to eat and when to go to bed. It aims to increase participation through communication and improve care planning [54]. The DCI explores the abilities of people living with dementia to control everyday decision-making preferences. The majority of the findings are however, about home based care identifying that care partners were chosen as primary substitute decision-makers, but financial strain was correlated with how well people living with dementia felt they were understood [24, 42, 49].

Decision-making participation characteristics were explored in a qualitative study of 21 care dyad interviews with older people living with dementia and their care partners [11]. Aspects explored were; information received, being listened to, ability to express an opinion, time allowed for reflection, and opportunity to change the decision. People living with dementia felt that they were not given enough time to reflect on decisions and did not feel their views about care provision were listened to. Care partners reported greater satisfaction with the quality of the communication and decision-making process [11].

Only one study explored the person living with dementia's *desire* to participate in decision-making [46]. Using a vignette, the study examined whether 48 people living with Alzheimer's disease would wish to participate in the decision to take a disease slowing medication, and what factors (including family) influenced their participation. In total 92% of people living with dementia wanted to participate in the decision, whilst only 71% of their care partners thought they would. People living with dementia concentrated on *involvement* in the *process*, whilst their care partners focused on their relatives' *capacity* to participate. Paradoxically, half of the people living with dementia that were formally assessed as lacking capacity (n=29) had care partners who said their relatives would be involved in decision-making [46].

For peer review only - http://bmjopen.bmj.com/site/about/guidelines.xhtml

Facilitators and inhibitors of shared decision-making

Care Partners and professionals

Where roles in decision-making are grounded in 'relationships' (or connectedness), the roles of care partners and workers can be facilitators or barriers [see e.g. 41, 44, 47, 52].

Only one study explicitly raised the question of who should participate in decision-making and what their roles should be [53]. Yet the impact of the relationship between the person living with dementia and their care partner on decision-making involvement is well documented [40, 46, 47, 49, 52]. There is little research focussing on shared decision-making relationships in extended care setting with the majority being undertaken in care dyads within the home environment [see e.g. 21, 22].

Clarke and colleagues [41] observed that care workers' characteristics could facilitate or inhibit decision-making involvement for people living with dementia in extended care settings. Positive characteristics included warmth, encouragement with memory, and routine. Routine also featured as a negative characteristic if linked to task orientated care. An example is given of a resident who appeared to be asleep and the carer had the person's feet out of bed before explaining what they intended to do [41 p20]. Other negative characteristics inhibiting shared decision-making were; discouraging independence, depersonalisation and risk adversity. The researchers observed more negative incidents than positive ones and felt that residents' autonomy was compromised [41]. Opportunities for expression of choice were reported to be reduced with the increase of daily care needs in extended care environments [45, 48]. Three studies identified reduced shared decision-making associated with; social attitudes, lack of available choices, systems, and care partners and workers identifying more problems than opportunities to involve the person living with dementia [10, 48, 53]. Optimal decision involvement was achieved by recognising the abilities and rights of the person living with a cognitive impairment as capable of influencing the decision, giving and sharing information, offering support and reinforcing opinions [10, 11, 40].

For peer review only - http://bmjopen.bmj.com/site/about/guidelines.xhtml

Tools and resources

Four studies developed and/or evaluated the use of tools and resources to facilitate shared decision-making for someone with cognitive impairment [39, 43, 51, 53]. Two studies evaluated the use of Talking Mats (TMs) which are a picture based, communication framework that allow people to indicate their feelings within a given topic by placing the relevant image on a visual scale. The first study compared TMs with structured and unstructured communication methods with five people in the late stages of Huntingdon's disease [43] and the second study compared TMs to usual communication methods with 18 people living with dementia and their care partners [51]. Both studies reported improvements in satisfaction with discussions when using TMs, although in one study [51] the feeling of involvement was significantly higher for the carers than for the people living with dementia.

Two studies developed computerised tools to support shared decision-making [39, 53]. Span and colleagues [53] co-designed an interactive web based tool to promote remote involvement of people living with dementia and their care partners in decision-making around such topics as; social contacts and daily activities, mobility and safety, future care, and finances [53]. Bailey and colleagues [39] created a visual aid for people living with a learning disability to facilitate participation in decision-making by presenting different types of information in a uniform format. They argue that this uniformity throughout a decision process could support everyday choices. The participants with a learning disability were trained to use the visual aid and their level of decision-making involvement improved as a result, although ongoing use was required to maintain the improvement [39].

Benefits of shared decision-making for people living with a cognitive impairment

Consulting residents living with dementia in an extended care environment, about the care home décor, Godwin [45] noted as an ancillary finding that residents appeared to be "surprised and pleased" to be asked [45 p. 114], arguing that this kind of consultation could enhance their self-esteem and contribute to their quality of life. Whilst this study did not measure the impact of the decision-making process, other studies have identified such benefits as heightened self-esteem, purpose and feeling of self-worth, as outcomes from retaining involvement in decision-making [44]. Ongoing decision-making involvement for

For peer review only - http://bmjopen.bmj.com/site/about/guidelines.xhtml

people living with dementia is also correlated with reduced depressive symptoms and maintained everyday functioning [24].

Discussion

The available evidence suggests that people living with cognitive impairment want the opportunity to participate in decision-making about their health and care; it can contribute to a sense of worth and has the potential to improve quality of life [11, 44, 46]. A lack of opportunity to participate in decision-making is a significant and consistent barrier throughout the literature reviewed [e.g. 10, 41, 48, 50]. This may be due to confusion about what shared decision-making is in everyday care for people living with cognitive impairment and whether it is in fact opportunities for greater choice that are required. Or it may be professionals' lack of skills in recognising and facilitating peoples' desire and ability to make a decision [50]. People living with dementia concentrated on *involvement* in the decision-making *process* [11, 44, 46] but there is a lack of evidence about how the person living with cognitive impairment chooses who they make decisions with and which resources or tools could facilitate people living with a cognitive impairment to *lead* the conversations.

Practical interventions to support and facilitate various aspects of the decision-making process (such as TMs and computer software tools) are reported as having good outcomes for the person living with a cognitive impairment, their care partner and in some cases their health or care professional too [39, 43, 51, 53]. However, current tools predominantly rely on the care partner or professional to identify the decision topic, potentially disempowering the person living with a cognitive impairment. In addition implementing shared decision-making resources in extended care environments would require care workers being given the time, resource and authority to develop the skills required to use such aids. The included studies fail to provide evidence or discussion of the cost implications associated with embedding shared decision-making for people living with cognitive impairments, or the staff development needed to implement everyday decision-making in extended care settings.

Whether the importance of interdependent relationship between the (family) care partner and the person living with dementia in facilitating or inhibiting shared decision-making at

For peer review only - http://bmjopen.bmj.com/site/about/guidelines.xhtml

home [40, 46, 47, 49, 52] is reflected by the relationship between the person living with dementia and their care staff in extended care settings is not yet understood. However, the frequent underestimation of care partners and workers of the desire and ability of people living with moderate and severe dementia to express preferences about their daily care [10, 11, 47, 48, 52] combined with the incongruence in levels of satisfaction in the decision-making process between care partners and people living with dementia [11, 46, 54] raises concerns about the role of consultees under the Mental Capacity Act (2005) [15] in the United Kingdom. Although as the process of decision-making may be as important as the decision itself [11, 42, 44, 45, 47], it could be argued that if all parties *perceive* that they have optimum levels of involvement and the desired outcomes are achieved the shared decision-making process is a success.

From the limited evidence available on how relationships in extended care affect shared decision-making it appears that how care staff engage is crucial. They can be enablers or blockers to shared decisions, and this appears to be related to their personality, communication skills and the routines of the workplace [41, 45, 50]. The implications of the relationships between people living with dementia in extended care and their care staff become increasingly significant as the dementia progresses with care needs increasing and communication capabilities dwindling. A greater understanding of the decision-making needs of the person living with dementia in extended care, and how they can be met, is therefore needed.

Strengths and Limitations

The review involved a systematic and rigorous search for literature relating to shared decision-making for people living with cognitive impairment in extended care settings. As such, this review provides a baseline to inform future research and practice. However, the majority of studies were conducted in the community rather than in extended care settings, quality was variable and there is little evidence on what supports the negotiation of day-to-day decisions between people with a cognitive impairment and their (staff and family) carers in extended care.

For peer review only - http://bmjopen.bmj.com/site/about/guidelines.xhtml

The review highlights the difficulties defining what is meant by shared decision-making for people who are cognitively impaired. It is recognised that terminology varies across Countries, disciplines and professions potentially impacting on the studies retrieved. Including additional search terms around 'choice' may have identified additional relevant papers that were not identified from terms relating to shared decision-making.

Quality assessment of qualitative studies was changed from the protocol [27] due to access issues so the QualSyst tool [35] was used. This tool has limitations particularly related to lack of requirement to assess an ethics statement.

Conclusion

Evidence suggests that people living with cognitive impairment value opportunities to be involved in everyday decision-making about their care although there remains some confusion about what constitutes shared decision-making in everyday care for people living with cognitive impairment in extended care. How to embed the process of shared decision-making into everyday practice in extended care and whether declining health and function are real or perceived barriers to participation remains to be determined along with the impact it would have on the quality of life of people living with dementia in care homes.

Further research is required to understand how opportunities for shared decision-making are created, recognised and understood; and whether they could improve the quality of life for people living with dementia in care homes. Research exploring the relationship between the person living with dementia and their care staff would improve understanding of how shared decision-making can be better facilitated in extended care environments.

Authors' contributions

RD wrote the protocol, undertook data extraction, quality assessment and analysis and wrote the paper. FB and CG supervised and critically appraised all aspects of the process. Important changes from the protocol have been explained. All authors read and approved the final manuscript.

For peer review only - http://bmjopen.bmj.com/site/about/guidelines.xhtml

Funding statement

This review is undertaken as part of a wider doctoral study focusing on dementia care in care homes which has been funded by the National Institute for Health Research (NIHR) Collaboration for Leadership in Applied Health Research & Care (CLAHRC) East of England.

This report presents independent research funded by the National Institute for Health Research (NIHR) Collaboration for Leadership in Applied Health Research & Care (CLAHRC) East of England, at Cambridgeshire and Peterborough NHS Foundation Trust. The views expressed are those of the author(s) and not necessarily those of the NHS, the NIHR or the Department of Health.

Competing Interests

Authors declare that they have no competing interests.

References

1. Edvardsson, D., D. Fetherstonhaugh, and R. Nay, *Promoting a continuation of self and normality: person-centred care as described by people with dementia, their family members and aged care staff.* Journal of clinical nursing, 2010. **19**(17-18): p. 2611-2618.

2. Hutchison, B., et al., *Primary health care in Canada: systems in motion.* Milbank Quarterly, 2011. **89**(2): p. 256-288.

3. Stiggelbout, A.M., et al., *Shared decision making: really putting patients at the centre of healthcare.* Bmj, 2012. **344**(S 28).

4. Wolfs, C.A., et al., *Rational decision-making about treatment and care in dementia: A contradiction in terms?* Patient Education & Counseling, 2012. **87**(1): p. 43-48 6p.

5. Edvardsson, D., B. Winblad, and P.-O. Sandman, *Person-centred care of people with severe Alzheimer's disease: current status and ways forward.* The Lancet Neurology, 2008. **7**(4): p. 362-367.

6. McCormack, B., *Person-centredness in gerontological nursing: an overview of the literature.* Journal of clinical nursing, 2004. **13**(s1): p. 31-38.

For peer review only - http://bmjopen.bmj.com/site/about/guidelines.xhtml

7. Bartlett, R. and D. O'Connor, *Broadening the dementia debate: Towards social citizenship*. 2010: Policy Press.

8. Wilkinson, H., *Empowerment and decision-making for people with dementia: the use of legal interventions in Scotland.* Aging Ment Health, 2001. **5**(4): p. 322-8.

9. Bekker, H.L., *Using decision-making theory to inform clinical practice.* Shared decision-making in health care: achieving evidence-based patient choice, 2009. **2**: p. 45-51.

10. Smebye, K.L.K., M.: Engedal, K., *How do persons with dementia participate in decision making related to health and daily care? a multi-case study.* BMC Health Serv Res, 2012. **12**: p. 241.

11. Tyrrell, J.G., N.: Myslinski, M., *Freedom of choice and decision-making in health and social care: views of older patients with early-stage dementia and their carers.* Dementia (14713012), 2006. **5**(4): p. 479-502 24p.

12. Nations, U., *Convention on the Rights of Persons with Disabilities (CRPD)*, U. Nations, Editor. 2006, Division for Social Policy and Development Disability: New York.

13. Smebye, K.L., M. Kirkevold, and K. Engedal, *How do persons with dementia participate in decision making related to health and daily care? A multi-case study.* BMC health services research, 2012. **12**(1): p. 241.

14. Manthorpe, J.S., K., *Changing practice: Adapting to the Mental Capacity Act 2005.* Social Care and Neurodisability, 2013. **4**(3): p. 124-133.

15. Affairs, D.o.C., *Mental Capacity Act 2005*, D.o.C. Affairs, Editor. 2005, HMSO London.

16. Affairs, D.o.C., *Mental Capacity Act 2005; code of practice.* , D.o.C. Affairs, Editor. 2007, TSO: London.

17. Boyle, G., *The Mental Capacity Act 2005: promoting the citizenship of people with dementia?* Health & Social Care in the Community, 2008. **16**(5): p. 529-537 9p.

18. Manthorpe, J.S., K., *Mental capacity and dementia: A review. Part 2.* Journal of Dementia Care, 2013. **21**(1): p. 35-38.

19. Goodman, C., et al., *Preferences and priorities for ongoing and end-of-life care: A qualitative study of older people with dementia resident in care homes.* International journal of nursing studies, 2013. **50**(12): p. 1639-1647.

For peer review only - http://bmjopen.bmj.com/site/about/guidelines.xhtml

20. Mathie, E., et al., *An uncertain future: the unchanging views of care home residents about living and dying.* Palliative Medicine, 2012. **26**(5): p. 734-743.

21. Miller, L.M., C.J. Whitlatch, and K.S. Lyons, *Shared decision-making in dementia: A review of patient and family carer involvement.* Dementia, 2014.

22. Larsson, A.T. and J.H. Österholm, *How are decisions on care services for people with dementia made and experienced? A systematic review and qualitative synthesis of recent empirical findings.* International Psychogeriatrics, 2014. **26**(11): p. 1849-1862.

23. Taghizadeh Larsson, A.O., J. H., *How are decisions on care services for people with dementia made and experienced? A systematic review and qualitative synthesis of recent empirical findings.* Int Psychogeriatr, 2014. **26**(11): p. 1849-62.

24. Menne, H.L.W., C. J., *Decision-making involvement of individuals with dementia.* Gerontologist, 2007. **47**(6): p. 810-9.

25. Mariani, E., et al., *Shared decision-making on a 'life-and-care plan'in long-term care facilities: research protocol.* Nursing Open, 2016.

26. Orrell, M., et al., *The needs of people with dementia in care homes: the perspectives of users, staff and family caregivers.* International Psychogeriatrics, 2008. **20**(05): p. 941-951.

27. Daly, R., F. Bunn, and C. Goodman, *Shared decision-making for people living with dementia in extended care settings: protocol for a systematic review.* BMJ open, 2016. **6**(11): p. e012955.

28. Moher, D., et al., *Preferred reporting items for systematic review and meta-analysis protocols (PRISMA-P) 2015 statement.* Syst Rev, 2015. **4**: p. 1.

29. Kitwood, T., *The experience of dementia.* Aging & Mental Health, 1997. **1**(1): p. 13-22.

30. Kitwood, T., *The concept of personhood and its relevance for a new culture of dementia care*. 1997: London: Routledge.

31. Kitwood, T., *Dementia Reconsidered*. 1997, Buckingham, UK: Open University Press.

32. CQC, C.Q.C., *Care Homes.* 2009.

33. Laidsaar-Powell, R., et al., *Physician–patient–companion communication and decision-making: a systematic review of triadic medical consultations.* Patient Education and Counseling, 2013. **91**(1): p. 3-13.

For peer review only - http://bmjopen.bmj.com/site/about/guidelines.xhtml

34. Légaré, F., et al., *Barriers and facilitators to implementing shared decision-making in clinical practice: Update of a systematic review of health professionals' perceptions.* Patient Education and Counseling, 2008. **73**(3): p. 526-535.

35. Kmet L, L.R., Cook L., *Standard Quality Assessment Criteria for Evaluating Primary Research Papers from a Variety of Fields*. 2004, Alberta Heritage Foundation for Medical Research: Edmonton.

36. Noyes J, H.K., Booth A, Harris J, Harden A, Popay J, Pearson A, Cargo M, and Pantoja T, *Qualitative research and Cochrane reviews. In:*, in *Cochrane Handbook for Systematic Reviews of Interventions* G.S. Higgins JPT, Editor. 2015, The Cochrane Collaboration: http://qim.cochrane.org/supplemental-handbook-guidance.

37. Hannes, K., *Critical appraisal of qualitative research*, in *Supplementary Guidance for Inclusion of Qualitative Research in Cochrane Systematic Reviews of Interventions.*, B.A. Noyes J, Hannes K, Harden A, Harris J, Lewin S, Lockwood C. , Editor. 2011, Cochrane Collaboration Qualitative Methods Group: http://cqrmg.cochrane.org/supplemental-handbook-guidance.

38. Braun, V. and V. Clarke, *Using thematic analysis in psychology.* Qualitative research in psychology, 2006. **3**(2): p. 77-101.

39. Bailey, R.W., P.: Dymond, S., *A visual aid to decision-making for people with intellectual disabilities.* Research in Developmental Disabilities, 2011. **32**(1): p. 37-46 10p.

40. Boyle, G., *Recognising the agency of people with dementia.* Disability & Society, 2014. **29**(7): p. 1130-1144 15p.

41. Clarke, A.M.D., M. F., *Communication and decision making among residents with dementia.* Geriatrician, 2004. **22**(3): p. 17-24 8p.

42. Feinberg, L.F.W., C. J., *Decision-making for persons with cognitive impairment and their family caregivers.* Am J Alzheimers Dis Other Demen, 2002. **17**(4): p. 237-44.

43. Ferm, U.S., A.: Sundin, L.: Hartelius, L., *Using Talking Mats to support communication in persons with Huntington's Disease.* International Journal of Language & Communication Disorders, 2010. **45**(5): p. 523-536 14p.

44. Fetherstonhaugh, D.T., L.: Nay, R., *Being central to decision making means I am still here!: the essence of decision making for people with dementia.* J Aging Stud, 2013. **27**(2): p. 143-50.

45. Godwin, B., *Colour consultation with dementia home residents and staff.* Quality in Ageing & Older Adults, 2014. **15**(2): p. 102-119 18p.

For peer review only - http://bmjopen.bmj.com/site/about/guidelines.xhtml

46. Hirschman, K.B.J., C. M.: James, B. D.: Xie, S. X.: Karlawish, J. H., *Do Alzheimer's disease patients want to participate in a treatment decision, and would their caregivers let them?* Gerontologist, 2005. **45**(3): p. 381-8.

47. Horton-Deutsch, S.T., Prudence: Evans, Rebecca, *Health care decision-making of persons with dementia.* Dementia, 2007. **6**(1): p. 105-120.

48. Kjellberg, A., *More or less independent.* Disability & Rehabilitation, 2002. **24**(16): p. 828-840 13p.

49. Menne, H.L.T., S. S.: Whitlatch, C. J.: Feinberg, L. F., *Decision-making involvement scale for individuals with dementia and family caregivers.* Am J Alzheimers Dis Other Demen, 2008. **23**(1): p. 23-9.

50. Milte, C.M.R., Julie: Davies, Owen: Whitehead, Craig: Masters, Stacey: Crotty, Maria, *Family meetings for older adults in intermediate care settings: the impact of patient cognitive impairment and other characteristics on shared decision making.* Health Expectations, 2015. **18**(5): p. 1030-1040.

51. Murphy, J.O., T., *The use of Talking Mats to support people with dementia and their carers to make decisions together.* Health Soc Care Community, 2013. **21**(2): p. 171-80.

52. Samsi, K. and J. Manthorpe, *Everyday decision-making in dementia: findings from a longitudinal interview study of people with dementia and family carers.* International Psychogeriatrics, 2013. **25**(6): p. 949-961 13p.

53. Span, M., *Developing an interactive web tool to facilitate shared decision-making in dementia care networks: a participatory journey*. 2016, Amsterdam: Vrije Universiteit.

54. Whitlatch, C.J.F., Lynn Friss Tucke, Shandra S., *Measuring the Values and Preferences for Everyday Care of Persons with Cognitive Impairment and Their Family Caregivers.* The Gerontologist, 2005. **45**(3): p. 370-380.

55. Elwyn, G., et al., *Investing in deliberation: a definition and classification of decision support interventions for people facing difficult health decisions.* Medical Decision Making, 2010. **30**(6): p. 701-711.

56. Feinberg, L.F. and J. Whitlatch, *Decision-making for persons with cognitive impairment and their family caregivers.* American journal of Alzheimer's disease and other dementias, 2002. **17**(4): p. 237-244.

57. Thompson, A.G., *The meaning of patient involvement and participation in health care consultations: a taxonomy.* Social science & medicine, 2007. **64**(6): p. 1297-1310.

For peer review only - http://bmjopen.bmj.com/site/about/guidelines.xhtml

For peer review only

For peer review only - http://bmjopen.bmj.com/site/about/guidelines.xhtml

PRISMA 2009 Flow Diagram

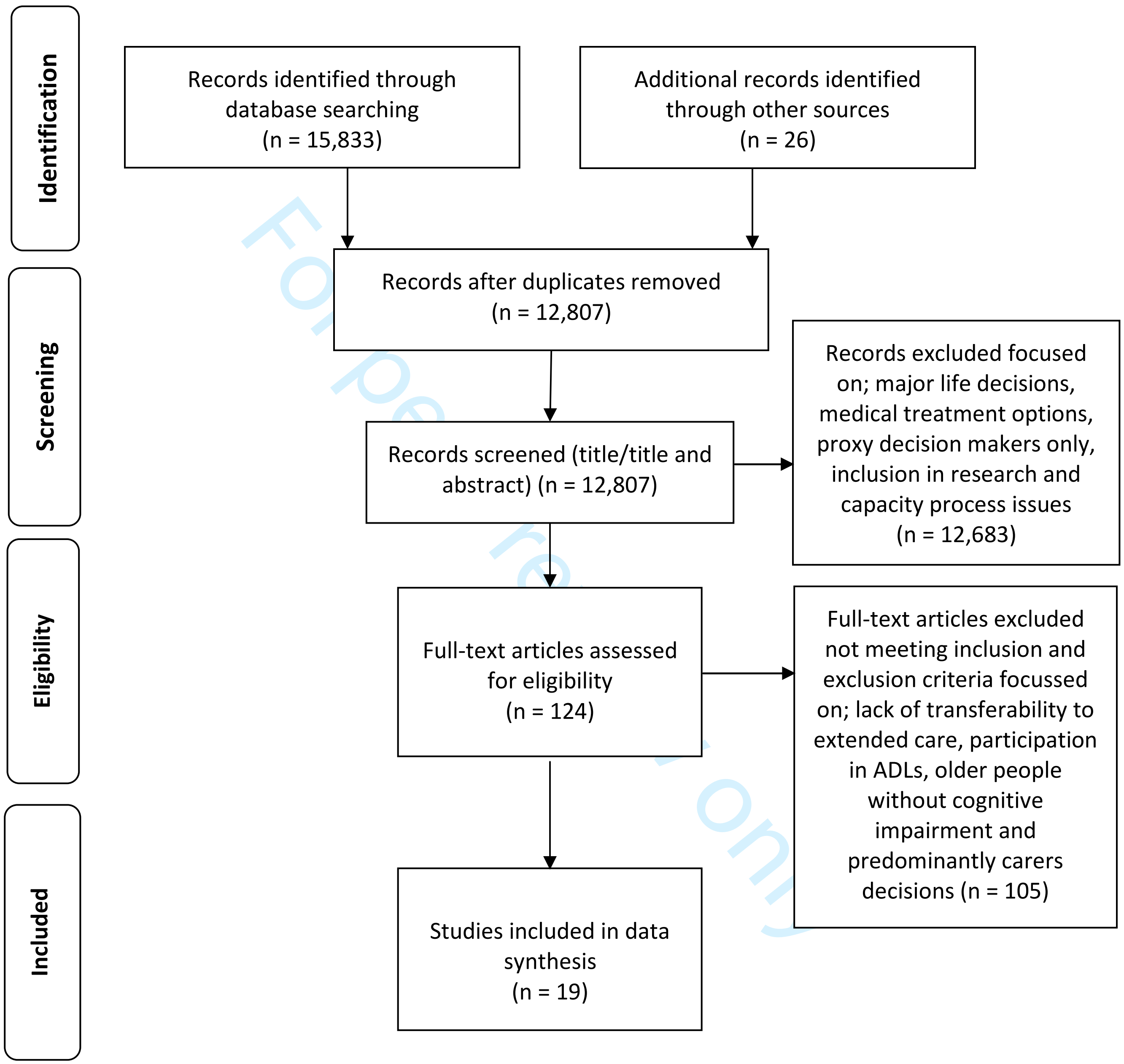

From: Moher D, Liberati A, Tetzlaff J, Altman DG, The PRISMA Group (2009). *P*referred *R*eporting *I*tems for *S*ystematic Reviews and *M*eta-*A*nalyses: The PRISMA Statement. PLoS Med 6(7): e1000097. doi:10.1371/journal.pmed1000097

For more information, visit www.prisma-statement.org.

For peer review only - http://bmjopen.bmj.com/site/about/guidelines.xhtml

PRISMA 2009 Checklist SHARED DECISION-MAKING FOR PEOPLE LIVING WITH COGNITIVE IMPAIRMENT IN EXTENDED CARE SETTINGS: A SYSTEMATIC REVIEW

Section/topic	#	Checklist item	Reported on page #
**TITLE**			
Title	1	Identify the report as a systematic review, meta-analysis, or both.	3,4
**ABSTRACT**			
Structured summary	2	Provide a structured summary including, as applicable: background; objectives; data sources; study eligibility criteria, participants, and interventions; study appraisal and synthesis methods; results; limitations; conclusions and implications of key findings; systematic review registration number.	2
**INTRODUCTION**			
Rationale	3	Describe the rationale for the review in the context of what is already known.	3-4
Objectives	4	Provide an explicit statement of questions being addressed with reference to participants, interventions, comparisons, outcomes, and study design (PICOS).	4-5
**METHODS**			
Protocol and registration	5	Indicate if a review protocol exists, if and where it can be accessed (e.g., Web address), and, if available, provide registration information including registration number.	4
Eligibility criteria	6	Specify study characteristics (e.g., PICOS, length of follow-up) and report characteristics (e.g., years considered, language, publication status) used as criteria for eligibility, giving rationale.	5
Information sources	7	Describe all information sources (e.g., databases with dates of coverage, contact with study authors to identify additional studies) in the search and date last searched.	6-7
Search	8	Present full electronic search strategy for at least one database, including any limits used, such that it could be repeated.	6-7
Study selection	9	State the process for selecting studies (i.e., screening, eligibility, included in systematic review, and, if applicable, included in the meta-analysis).	8
Data collection process	10	Describe method of data extraction from reports (e.g., piloted forms, independently, in duplicate) and any processes for obtaining and confirming data from investigators.	8
Data items	11	List and define all variables for which data were sought (e.g., PICOS, funding sources) and any assumptions and simplifications made.	5
Risk of bias in individual studies	12	Describe methods used for assessing risk of bias of individual studies (including specification of whether this was done at the study or outcome level), and how this information is to be used in any data synthesis.	8
Summary measures	13	State the principal summary measures (e.g., risk ratio, difference in means).	8
Synthesis of results	14	Describe the methods of handling data and combining results of studies, if done, including measures of consistency (e.g., I^2) for each meta-analysis.	8

For peer review only - http://bmjopen.bmj.com/site/about/guidelines.xhtml

PRISMA 2009 Checklist SHARED DECISION-MAKING FOR PEOPLE LIVING WITH COGNITIVE IMPAIRMENT IN EXTENDED CARE SETTINGS: A SYSTEMATIC REVIEW

Section/topic	#	Checklist item	Reported on page #
Risk of bias across studies	15	Specify any assessment of risk of bias that may affect the cumulative evidence (e.g., publication bias, selective reporting within studies).	8
Additional analyses	16	Describe methods of additional analyses (e.g., sensitivity or subgroup analyses, meta-regression), if done, indicating which were pre-specified.	8
**RESULTS**			
Study selection	17	Give numbers of studies screened, assessed for eligibility, and included in the review, with reasons for exclusions at each stage, ideally with a flow diagram.	8-10
Study characteristics	18	For each study, present characteristics for which data were extracted (e.g., study size, PICOS, follow-up period) and provide the citations.	10
Risk of bias within studies	19	Present data on risk of bias of each study and, if available, any outcome level assessment (see item 12).	9
Results of individual studies	20	For all outcomes considered (benefits or harms), present, for each study: (a) simple summary data for each intervention group (b) effect estimates and confidence intervals, ideally with a forest plot.	11-17
Synthesis of results	21	Present results of each meta-analysis done, including confidence intervals and measures of consistency.	11
Risk of bias across studies	22	Present results of any assessment of risk of bias across studies (see Item 15).	9
Additional analysis	23	Give results of additional analyses, if done (e.g., sensitivity or subgroup analyses, meta-regression [see Item 16]).	10
**DISCUSSION**			
Summary of evidence	24	Summarize the main findings including the strength of evidence for each main outcome; consider their relevance to key groups (e.g., healthcare providers, users, and policy makers).	17-18
Limitations	25	Discuss limitations at study and outcome level (e.g., risk of bias), and at review-level (e.g., incomplete retrieval of identified research, reporting bias).	18
Conclusions	26	Provide a general interpretation of the results in the context of other evidence, and implications for future research.	19
**FUNDING**			
Funding	27	Describe sources of funding for the systematic review and other support (e.g., supply of data); role of funders for the systematic review.	20

From: Moher D, Liberati A, Tetzlaff J, Altman DG, The PRISMA Group (2009). Preferred Reporting Items for Systematic Reviews and Meta-Analyses: The PRISMA Statement. PLoS Med 6(7): e1000097. doi:10.1371/journal.pmed1000097

For more information, visit: **www.prisma-statement.org**.

For peer review only - http://bmjopen.bmj.com/site/about/guidelines.xhtml

BMJ Open

SHARED DECISION-MAKING FOR PEOPLE LIVING WITH DEMANTIA IN EXTENDED CARE SETTINGS: A SYSTEMATIC REVIEW

Journal:	*BMJ Open*
Manuscript ID	bmjopen-2017-018977.R1
Article Type:	Research
Date Submitted by the Author:	22-Feb-2018
Complete List of Authors:	Daly, Rachel; University of Hertfordshire, CRIPACC<br>Bunn, Frances; University of Herfordshire, Department of Health and Human Sciences<br>Goodman, Claire; University of Hertfordshire, Centre for Research in Primary and Community Care
**Primary Subject Heading**:	Evidence based practice
Secondary Subject Heading:	Mental health, Communication, Geriatric medicine, Patient-centred medicine
Keywords:	Dementia < NEUROLOGY, Delirium & cognitive disorders < PSYCHIATRY, shared decision-making, Care Homes, Extended care, Everyday care

SCHOLARONE™
Manuscripts

For peer review only

Title: SHARED DECISION-MAKING FOR PEOPLE LIVING WITH DEMENTIA IN EXTENDED CARE SETTINGS: A SYSTEMATIC REVIEW.

Authors:

Rachel Daly, Queens Nurse, NIHR RDCP programme CLAHRC East of England doctoral student r.daly2@herts.ac.uk

Centre for Research in Primary and Community Care (CRIPACC), University of Hertfordshire, College Lane, Hatfield, Herts, England, AL10 9AB

Professor Frances Bunn, Professor in Health and Complex Conditions f.bunn@herts.ac.uk

Centre for Research in Primary and Community Care (CRIPACC), University of Hertfordshire, College Lane, Hatfield, Herts, England, AL10 9AB

Professor Claire Goodman, Professor Health Services Research c.goodman@herts.ac.uk

Centre for Research in Primary and Community Care (CRIPACC), University of Hertfordshire, College Lane, Hatfield, Herts, England, AL10 9AB

Corresponding author Rachel Daly – r.daly2@herts.ac.uk Tel: +44 7967 761298

Keywords: Cognitive Impairment, Dementia, Shared Decision-Making, Care Homes, Everyday Care, Extended Care, Day-to-Day Care, Residential Care

Tables – 3

References – 59

Supplementary files – Supplementary Figure 1 (Prisma flow chart)

For peer review only - http://bmjopen.bmj.com/site/about/guidelines.xhtml

ABSTRACT

Background – Shared decision-making is recognized as an important element of person-centered dementia care.

Objectives - The aim of this review was to explore how people living with dementia and cognitive impairment can be included in day-to-day decisions about their health and care in extended care settings

Design – A systematic review including primary research relating to shared decision-making, with cognitively impaired adults in (or transferrable to) extended care settings. Databases searched were: CINAHL, PubMed, the Cochrane Library, NICE Evidence, OpenGrey, Autism Data, Google Scholar, Scopus and Medicines Complete (June - October 2016 and updated 2018) for studies published in the last 20 years. PROSPERO number CRD42016035919

Results. Of the 19 included studies 15 involved people with living dementia, 7 in extended care settings. People living with cognitive impairment often have the desire and ability to participate in decision-making about their everyday care, although this is regularly underestimated by their staff and family care partners. Shared decision-making has the potential to improve quality of life for both the person living with dementia and those who support them. How resources to support shared decision-making are implemented in extended care settings, is less well understood.

Conclusions – Evidence suggests that people living with cognitive impairment value opportunities to be involved in everyday decision-making about their care. How these opportunities are created, understood, supported and sustained in extended care settings remains to be determined.

Strengths and limitations;

- The review involved a systematic and rigorous search for cross-disciplinary literature relating to shared decision-making for people living with cognitive impairment in extended care settings.
- The majority of studies were conducted in the community rather than in extended care settings.

For peer review only - http://bmjopen.bmj.com/site/about/guidelines.xhtml

- Terminology varies across Countries, disciplines and professions potentially impacting on the studies retrieved. Including additional search terms around 'choice' may have identified additional relevant papers.

Word Count – 4,571

SHARED DECISION-MAKING FOR PEOPLE LIVING WITH DEMENTIA IN EXTENDED CARE SETTINGS: A SYSTEMATIC REVIEW.

Background

For people living with dementia shared decision-making is increasingly considered crucial in health and care practice [1-4] and is an essential aspect of person centred care [5];the fundamental premise of which is that the individual's priorities, interests, abilities and character should inform decision-making [6]. Shared decision-making practice is equally important in extended care. Extended care is defined as residential settings that provide onsite care. This includes supported living, care villages and extra care housing in addition to more traditional care homes with and without nursing [7].

A dementia diagnosis does not automatically render someone incapable of making a decision [8, 9] but for a decision to truly be shared, mutuality must be established [10]. Legislative changes in the last decade have strengthened the rights of people living with dementia to participate in decisions about their care [11, 12]. The United Nations Convention on the Rights of People with Disabilities (CRPD), states that "persons with disabilities enjoy legal capacity on an equal basis with others in all aspects of life" [13]. However, evidence on *how* care decisions are currently made within various legislative frameworks, for and with, people living with dementia, is patchy and often focuses on decisions relating to life events [11, 14]. In England and Wales the Mental Capacity Act (2005) provides a legislative framework to protect and empower people to participate in decisions about their life and care unless there is evidence to the contrary [15, 16]. Evidence suggests however, that staff in extended care often make day-to-day care decisions on behalf of people living with dementia [17, 18]. There are situations where people living with dementia in extended care settings decline to participate in decision-making and prefer to relinquish control and delegate to a care partner or worker. Reasons identified include;

For peer review only - http://bmjopen.bmj.com/site/about/guidelines.xhtml

anxiety about ability to participate and reluctance to accept decreasing abilities (reported as self-protection) [see e.g. 19, 20, 21].

Two reviews have focused on shared decision-making with people living at home with dementia [21, 22]. They offer useful insight into degrees of involvement in the decision-making process, what influences that involvement and how people participate [21, 22].In a review of 36 studies of people living with dementia and their carers Miller [21] identified assorted patterns of decision-making ranging from 'being free to make a choice' through 'supported autonomy' to 'being reliant upon carers'. Larsson's review of 24 studies on community decisions relating to access to care services developed three themes to capture how people living with dementia experience involvement in care decisions; *excluded, prior preferences taken into account,* and *current preferences respected* [23]. These reviews usefully bring shared decision-making with people living with dementia into focus but largely excluded people living in extended care settings, who are typically frailer and further along the dementia trajectory. They highlight that decisions for people living with dementia at home invariably involved a care partner/family member. However, family members are often unavailable for many of the day-to-day decisions undertaken in extended care due to time and geographical constraints.

There is increasing recognition that day-to-day decisions are potentially more significant in everyday quality of life than the more noteworthy issues such as treatment decisions or relocation [see e.g. 24, 25, 26]. Building on previous work, this paper considers the evidence on *how* day-to-day decisions are understood and negotiated between people with a cognitive impairment and their (staff and family) carers in extended care settings. The review objectives are presented in *Box 1.*

**BOX 1 – Review Objectives**

- Explore how shared decision making is understood and/or characterised for people living with dementia and their (staff and family) carers
- Explore the role of (staff and family) carers of people living with dementia in shared decision making care dyads
- Analyse identified risks and benefits associated with shared decision making for people with cognitive impairment
- Ascertain empirical evidence for the effectiveness of available shared decision making resources for people living with dementia
- Seek to understand the barriers and facilitators to effective shared decision making for people living with dementia and their (staff and family) carers
- Explore the extent to which shared decision making has been researched in extended care settings
- Identify implications for shared decision making in dementia care practice, policy and future research

For peer review only - http://bmjopen.bmj.com/site/about/guidelines.xhtml

Methods

A detailed account to the review methods are published elsewhere [27] PROSPERO registration number CRD42016035919. The review was planned and is reported in accordance with the Preferred Reporting Items for Systematic Reviews and Meta-Analyses (PRISMA) guidelines [28].

Results are presented to reflect relevance to the review question and objectives rather than frequency that the topic arises in the data and must represent a pattern across more than one included study to constitute a theme [29].

Study selection and inclusion criteria

Inclusion criteria are presented in *Table 1* below. A time limit of 20 years was applied to reflect the rapidly changing literature on involving people living with dementia in their care, published since the seminal works of Tom Kitwood [30-32]. The main focus of the review was on people with dementia but studies relating to adults with other types of cognitive impairment were also included because of the potential for transferable learning.

**Table 1 - Inclusion Criteria**	
**Publication Language**	English
**Publication Dates**	January 1996 - October 2016
**Target Population**	Adults, aged over 18 years, with any type of cognitive impairment for example (but not limited to) dementia, learning disabilities, Parkinson's and Huntington's disease
**Study Setting**	Community living at home or extended care settings e.g. supported living, or residential care. Studies must be considered transferable to people living with a cognitive impairment in an extended care setting (for example the person living with a cognitive impairment must be in receipt of care in addition to that provided by a family care partner and the resource should be suitable for implementation in extended care settings.

For peer review only - http://bmjopen.bmj.com/site/about/guidelines.xhtml

**Study Types**	All research designs including quantitative and qualitative research.
**Primary Outcomes**	Any outcome relating to involvement in care planning, satisfaction with decision (e.g. care was congruent with expressed choice), quality of life or wellbeing, and behavioural changes (e.g. reduction in distress)
**Additional Outcomes**	Family care partner and/or health or care professional satisfaction, and any documented adverse effects (e.g. falls, weight loss, adverse outcomes related to medication management).

Table 1–study inclusion criteria

The term 'care partner' has been adopted to represent a family member or friend who has a caring relationship with the person living with a cognitive impairment. The terms health and/or care professionals, staff or workers are used interchangeably to describe people who are paid to provide health or social care.

Search strategy

The search strategy drew on a range of cross discipline data sources associated with cognitive impairment and dementia care. Electronic databases searched and an example of the search query for PubMed is given in *table 2*. Search terms were adapted as appropriate for other databases.

**Table 2 - Search Strategy**	
**Electronic databases searched**	CINAHL Plus, PubMed, the Cochrane Library, NICE Evidence, OpenGrey, Autism Data, Google Scholar, Scopus and MedicinesComplete
**MeSH search terms (with subheadings included)**	Cognition Disorders searched (((("dementia"[MeSH Terms] OR "dementia"[All Fields]) OR ("cognitive dysfunction"[MeSH Terms] OR ("cognitive"[All Fields] AND "dysfunction"[All Fields]) OR "cognitive dysfunction"[All Fields] OR ("cognitive"[All Fields] AND "impairment"[All Fields]) OR "cognitive impairment"[All Fields])) OR ("learning disorders"[MeSH Terms] OR ("learning"[All Fields] AND "disorders"[All Fields]) OR "learning disorders"[All Fields] OR ("learning"[All Fields] AND "disability"[All Fields]) OR "learning disability"[All Fields])) OR ("learning disorders"[MeSH Terms] OR ("learning"[All Fields] AND "disorders"[All Fields]) OR "learning disorders"[All Fields] OR ("learning"[All Fields] AND "disorder"[All Fields]) OR "learning disorder"[All Fields])) OR ("autistic disorder"[MeSH Terms] OR

For peer review only - http://bmjopen.bmj.com/site/about/guidelines.xhtml

	("autistic"[All Fields] AND "disorder"[All Fields]) OR "autistic disorder"[All Fields] OR "autism"[All Fields])) OR ("stroke"[MeSH Terms] OR "stroke"[All Fields])) OR ("brain injuries"[MeSH Terms] OR ("brain"[All Fields] AND "injuries"[All Fields]) OR "brain injuries"[All Fields] OR ("brain"[All Fields] AND "injury"[All Fields]) OR "brain injury"[All Fields])) OR ("neurocognitive disorders"[MeSH Terms] OR ("neurocognitive"[All Fields] AND "disorders"[All Fields]) OR "neurocognitive disorders"[All Fields])) OR ("alzheimer disease"[MeSH Terms] OR ("alzheimer"[All Fields] AND "disease"[All Fields]) OR "alzheimer disease"[All Fields] OR "alzheimer"[All Fields])) AND Shared Decision-making ((((((shared[All Fields] AND ("Decision (Wash D C)"[Journal] OR "decision"[All Fields])) OR ("decision-making"[MeSH Terms] OR ("decision"[All Fields] AND "making"[All Fields]) OR "decision-making"[All Fields])) OR ("community participation"[MeSH Terms] OR ("community"[All Fields] AND "participation"[All Fields]) OR "community participation"[All Fields] OR ("consumer"[All Fields] AND "participation"[All Fields]) OR "consumer participation"[All Fields])) OR ("patient participation"[MeSH Terms] OR ("patient"[All Fields] AND "participation"[All Fields]) OR "patient participation"[All Fields])) OR (("Decision (Wash D C)"[Journal] OR "decision"[All Fields]) AND support[All Fields])) OR (care[All Fields] AND dyad[All Fields])) OR ("patient education handout"[Publication Type] OR "patient education as topic"[MeSH Terms] OR "patient education"[All Fields]))) NOT (((advance[All Fields] AND ("Decision (Wash D C)"[Journal] OR "decision"[All Fields])) OR ("advance directives"[MeSH Terms] OR ("advance"[All Fields] AND "directives"[All Fields]) OR "advance directives"[All Fields] OR ("advance"[All Fields] AND "directive"[All Fields]) OR "advance directive"[All Fields])) OR (advance[All Fields] AND care[All Fields] AND plan[All Fields]))) OR ((((((("Dementia"[Mesh] OR "Neurocognitive Disorders"[Mesh]) OR "Brain Injuries"[Mesh]) OR "Stroke"[Mesh]) OR "Learning Disorders"[Mesh]) OR "Autistic Disorder"[Mesh]) AND ((((("Decision-making"[Mesh] OR "Decision Support Techniques"[Mesh]) AND "Patient Participation"[Mesh]) OR "Cooperative Behavior"[Mesh]) OR "Physician-Patient Relations"[Mesh]) OR "Patient Education as Topic"[Mesh])) NOT Advance Directives("Advance Directives"[Mesh] OR "Advance Care Planning"[Mesh]))) NOT Paediatrics ("child"[MeSH Terms] OR "child"[All Fields])) NOT ("Child"[Mesh] OR "Disabled Children"[Mesh])
**Alternate free text search terms**	(Cogniti*, Disorder*, Dementia*, Alzheimer*, Neurocogniti* Dis*, Brain Injur*, Autis*, Learning Dis*, Stroke) AND (Shared Decision-making, Deci* Mak*, Patient Participat*, Consumer Participat* , Cooperat*, Decision Support) NOT (Paed* Child*) NOT (Advance Directives, Advance* care planning, Advance* deci*)
**Google Scholar**	The search and screening strategy for Google Scholar was agreed by all 3 authors. Free text search terms mirrored other databases. Results were filtered by relevance. The first 20 pages of results, title and abstract, were screened (20 results per page).

Table 2- Electronic databases searched, and search terms used

For peer review only - http://bmjopen.bmj.com/site/about/guidelines.xhtml

Reference lists of relevant primary and review articles were manually searched to detect additional studies and citation search was performed using the 'cited by' option on Google Scholar, and the 'related articles' option in PubMed. Searches were initially undertaken in June 2016 and updated in October 2016. Lateral searches were completed in November 2016. The search was updated in January 2018.

Electronic search results were downloaded into EndNote. One author (RD) screened references and, where necessary, sought support and independent review from a second author (FB or CG).

Quality Assessment and Data Extraction

Quality assessment was undertaken using the validated QualSyst Framework [33-35]. The QualSyst framework provides comprehensive definitions, instructions, and a scoring system for quality scoring of both quantitative and qualitative studies represented as a percentage (with a greater percentage representing higher quality)[35]. An ethical approval question was added as this has been highlighted as an essential element of study quality [36, 37]. Quality assessment was carried out by one reviewer (RD), with 10% checked by a second reviewer (FB/CG).

Data were extracted using a structured form that addressed the review objectives. This included information about the study design, participants and outcomes.

Analysis

Due to heterogeneity, and low numbers, of included quantitative studies, meta-analysis was not considered appropriate therefore results are reported in a narrative format.

Theoretical thematic analysis was undertaken using the research question and review objectives (see *box 1*) as a framework to map the range of data and identify recurrent themes. This method of synthesising qualitative research draws on work by Braun and Clarke [29, 38]. It offers in depth exploration of the identified themes and areas of interest, which include the roles, resources, and people essential to the shared decision-making process relevant to extended care settings.

For peer review only - http://bmjopen.bmj.com/site/about/guidelines.xhtml

Results

Nineteen publications are included [11, 12, 24, 39-54](see *table 3*) relating to 18 unique studies (including a doctoral dissertation). Four of the papers are linked as they included a baseline sample drawn from the same pool of participants [24, 42, 49, 54]. However, the studies addressed different questions and are, therefore, included as individual papers. An overview of the screening and selection process is demonstrated in supplementary Figure 1.

Supplementary Figure 1 - PRISMA flow chart

Study Quality

Study quality ranged from 65-100% see *table 3*. No studies were excluded as a result of the quality assessment but a high risk of bias was noted in some studies, such as a measure designed by the same people as the intervention [43]. Eight studies did not provide a clear ethics statement [12, 24, 41-44, 51, 54], including the two studies that scored 100% on QualSyst [49, 54].

Characteristics of included studies and participants

All included studies (see *table 3*) were published between 2002 and 2016. The majority (n=14) of studies were published in the last 10 years, suggesting an increasing awareness and interest in shared decision-making for people living with a cognitive impairment that reflects the progression in national and international legislation. All five UK studies were published since the full implementation of the Mental Capacity Act [15].

Most papers (n=15) focus on people living with dementia [11, 12, 24, 40-42, 44-47, 49, 51-54]; twelve are in 'care dyads' (with a care partner) or 'triads' (with a care partner and a health or care worker). Two papers centre on people with a learning disability [39, 48], one on people living with Huntington's disease [43] , and one includes some people with cognitive impairment [50]. Seven studies represent some participants living in extended care settings [11, 12, 41, 45, 46, 48, 50, 53], whilst the majority (n=12) relate to people living at home. Of

For peer review only - http://bmjopen.bmj.com/site/about/guidelines.xhtml

For peer review only

the 19 studies included, only two studies specified *shared decision-making in extended care settings* as their explicit focus [41, 45].

The majority of studies (n=15) employed interviews and/or observations (see *table 3*). Three of the linked studies used structured interviews and compared the views of people living with a cognitive impairment and their care partners [42, 49, 54]. One study, used structured observations of family meetings [50]. Three studies undertook ethnographic observations of people living with dementia within a care dyad or triad [11, 40, 41]. Four studies identified person centred care as a theoretical framework [40, 45, 51, 53].

A breakdown of whether studies aimed to understand, evaluate interventions or measure shared decision-making is presented in *table 3* and discussed within the narrative below. Results are presented in cross-cutting themes that explore decision-making participation or involvement for people living with a cognitive impairment in terms of; how shared decision-making is understood and how participation in decision-making is measured, facilitated and inhibited.

Results are presented to reflect relevance to the review question and objectives rather than frequency that the topic arises in the data [29].

For peer review only

Table 3 - Characteristics of included studies

Author(s) (year) and [reference No.]	Purpose of study	Country	Methods	Quality score	Setting	Participants	Sample size				Results or Outcomes
							PLWCI	CP	CW	Total	
**Clarke, A. M. (2004) [41]**	To influence SDM	Australia 3	Qualitative Interviews and observations	75%	extended care	PLWD and CW	13		13	26	4 positive and 5 negative carer characteristics were identified that impacted on decision-making.
**Fetherston-haugh, D. (2013) [44]**	To understand SDM		Qualitative Interviews	80%	home	PLWD		6		6	3 pairs of conflicting attributes identified: 1) subtle support versus taking over; 2) hanging on versus letting go; and 3) being central versus being excluded
**Milte, C. (2015) [50]**	To understand SDM		Quantitative Observed family meetings	83%	intermediate care	Older people, CPs and HCPs	51	51	2	104	Geriatricians' performance in SDM was mixed; above baseline skill level in some areas and below in others.<br>Longer meetings = better SDM by clinicians.
**Tyrrell, J. (2006) [12]**	To measure SDM	France 1	Qualitative Interviews	83%	home	PLWD and care partner	21	21		42	PLWD did not feel listened to and had limited freedom to participate in decision-making.<br>Carers were more satisfied than PLWD
**Span, M. (2016) [53]**	To facilitate SDM	Holland 1	Qualitative Interviews, focus groups, specialist consultation and workshops	75%	home	PLWD, CPs and HCPs				84	18 topics of problems and 8 topics addressing decision-making in emerged. Only 8 topics were identified by both PLWD and Care partners
**Smebye, K. L. (2012) [11]**	To understand SDM	Norway 1	Mixed methods Interviews and observations	95%	home and extended care	PLWD, CPs and HCPs	10	10	10	30	Care staff do not base mental competence on standardized tests; values and relationships as important as logic.<br>New decision-making categories emerged.<br>Autonomous decision-making occurred but SDM was most typical.
**Ferm, U. (2010) [43]**	To facilitate SDM	Sweden 2	Mixed methods Interviews	95%	home	PLW HD	5			5	Talking Mats increased communication but effectiveness depended on conversation topic.

For peer review only

For peer review only - http://bmjopen.bmj.com/site/about/guidelines.xhtml

**Kjellberg, A. (2002) [48]**	To understand		Qualitative Interviews	70%	home, extended and day care	People living with LD	23			23	Of the 9 theoretical combinations of levels of decision-making identified only 5 emerged.
**Bailey, R. (2011) [39]**	To measure SDM	United Kingdom 5	Quantitative Electronic decision-making tasks and questionnaire	79%	Day services	People living with LD	24			24	Decision-making task performances improved when using the visual aid designed. Although not sustained without the visual aid the improvement was regained when the aid and was reintroduced.
**Boyle, G. (2014) [40]**	To measure SDM		Qualitative Creative interaction, observation and interviews	85%	home	PLWD and CPs	5	5		10	Identified that agency related to SDM is demonstrated within six relevant themes
**Godwin, B. (2014) [45]**	To facilitate SDM		Mixed methods Consultation	90%	extended care	PLWD	34		42	76	Residents were able to demonstrate preferences relating to their environment and enjoyed the consultation process.
**Murphy, J. (2013) [51]**	To facilitate SDM		Mixed methods Researcher facilitated discussion	65%	home	PLWD and CPs	18	18		18	Participants felt more involved in discussions when using Talking Mats although feeling of involvement was significantly higher for carers than for PWLD
**Samsi, K. (2013) [52]**	To understand SDM		Qualitative Interviews	90%	home	PLWD and CPs	15	15		30	3 underlying principles identified of decision-making is negotiated and how dynamics changed: Importance of autonomy, decision specific approach and made on someone's behalf described as' best interest'.
**Feinberg, L. F. (2002) [42]**	To measure SDM	United States of America 6	Quantitative Interviews	80%	home	PLWD and CPs	51	51		102	Lower income and carer financial strain correlated with how well the PLWD felt their carer knew their care wishes (more financial strain = less understanding)
**Hirschman, K. B. (2005)[46]**	To understand SDM		Interviews	70%	Home and extended care	PLWD and CPs	48	48		96	Spousal carer - wife (90%) vs husband (21%). Half care partners of PLWD formally 'lacking

For peer review only - http://bmjopen.bmj.com/site/about/guidelines.xhtml

											capacity' still involved them in decision-making.
**Horton-Deutsch, S. (2007) [47]**	To understand SDM		Mixed methods Interviews	85%	home	PLWD and CPs	20	20		40	75% PLWD had always involved HCP and/or spouse in decisions. 50% PLWD decisions changed 80% in line with CP wishes. Only 55% of dyads congruent throughout. 20% PLWD maintained choice.
**Menne, H. L. (2008) [49]**	To measure SDM		Quantitative Interviews Demographic information inc. MMSE scores Capacity evaluation	100 %	home	PLWD and CPs	217	217		434	PLWD consistently considered themselves to have more involvement in decision-making than their care partners perceived them to be
**Menne, H. L. (2007) [24]**	To measure SDM		Quantitative Secondary data analysis	86%	home	PLWD and CPs	215	215		430	Greater decision-making involvement associated with younger, female, educated, non-spousal CP, fewer months since diagnosis, fewer problems with ADLs, fewer depressive symptoms, and place more importance on autonomy/self-identity
**Whitlatch, C. (2005) [54]**	To measure SDM		Mixed methods Interviews	100 %	home	PLWD and CPs	111	111		222	Values and preferences correlated with CP perceptions of PLWD quality of life and involvement in decision-making and with PLWD perception of own quality of life and involvement in decision-making.

HD – Huntington's Disease, SDM – shared decision-making, PLWCI – person living with cognitive impairment, PLWD – person living with dementia, LD – Learning disability CP – care partner, CW –care worker, HCP – health care professional

Table 3 - Characteristics of included studies

For peer review only

Understanding ‘shared decision-making’ for people living with cognitive impairment

There was no common understanding of what shared decision-making entails and how it can be operationalised for people living with a cognitive impairment. Only one paper offered a definition of shared decision-making. Defining it as an approach that involves patients in making medical decisions with their clinician [55], is influenced by the type and complexity of the decision being made and the perspectives of care partners as well as the person living with cognitive impairment.

Three ways that people living with dementia understand shared decision-making were identified in a phenomenological study [44]; subtle support versus taking over; hanging on versus letting go; and being central versus being marginalised or excluded. One participant described their negative response to being excluded;

> *“…if someone came in and started telling me how I should run things or do things, I think I would certainly retaliate and not conform to anything they would want to do.”* [44 p.147].

A recurrent theme in the qualitative literature reviewed is that for many people living with dementia, it is the participation or ‘sharing’ in the decision-making process, that is as, (if not more) important than making the decision itself [12, 42, 44, 47]. Despite this Samsi and Manthorpe [52] identified examples of ‘substituted decision-making’ that essentially excluded the person living with dementia;

> *“Oh I don't ask her what she wants anymore. I know what she'll say anyway - ‘anything you like, you decide’, so I just do what's best for us both.”* [52 p958]

Participation in decision-making

The extent to which a person living with dementia is able to participate in decision-making was a focus of many of the included studies. For the purpose of this paper participation in decision-making is subdivided into the degree or level of participation, ability to participate and desire to participate.

For peer review only - http://bmjopen.bmj.com/site/about/guidelines.xhtml

Levels of participation in decision-making

Five studies addressed levels of participation [11, 12, 47, 48, 52]. Smebye and colleagues used Thompson's five point taxonomy of patient involvement and participation in health care consultations [56]. Thompsons taxonomy ranges from entirely passive 'non-involvement', through cooperative 'shared decision-making', to independent 'autonomous decision-making' [56]. Smebye identified two additional elements through care triad observations and interviews; pseudo-autonomous, (assumptions about a person's decision) and delegated (the person living with dementia expressly delegates their decision-making) when including people living with dementia in care triads [11]. This extended taxonomy is reflected in all five studies exploring the extent of decision-making participation.

Shared-decision-making was considered as the most common decision-making pattern by people living with a cognitive impairment. Horton-Deutsch and colleagues [47] explored self-reported participation in decision-making for 20 people living with dementia and their care partners' using semi-structured interviews and a five point decision making scale centred on a treatment vignette. The scale ranged from (1) made decisions alone in the past with little input from others and continue to do so, to (5) discussed decisions with spouse in the past and continues to do so. Participants reported that although their self-perceived decision-making changed over time from, largely independent historically towards current interdependent (shared) decision-making, the majority (75%) of participants described some level of shared decision-making throughout [47]. Similarly, in a group of 23 people living with varying levels of learning disability, 70% rarely considered themselves entirely independent and relied on shared decision-making in some or all areas of their life [48].

Ability to participate

Seven studies [17, 39, 41, 42, 46, 49, 54] explored individuals' ability to participate in decisions often in the context of facilitators or barriers [e.g. 41]. All the studies presenting measures of decision-making participation compared the responses of the person living with a cognitive impairment with their care partners. They highlighted consistent incongruence between responses, with care partners typically believing the person living with dementia to be less involved than they perceived themselves [46, 54].

For peer review only - http://bmjopen.bmj.com/site/about/guidelines.xhtml

Five papers describe and/or evaluate tools that measure aspects of decision-making participation from the perspective of the person living with a cognitive impairment [12, 42, 44, 45, 47]. The linked studies (with an overlapping sample ranging from 51-217 care dyads) develop, use and evaluate the Decision Control Inventory (DCI) and the Decision-Making Involvement Scale (DMI) to assess and compare everyday care choices of both the person living with a cognitive impairment and their care partner [24, 42, 49, 54]. The DMI Scale measures involvement in everyday decisions such as what food to eat and when to go to bed. It aims to increase participation through communication and improve care planning [54]. The DCI explores the abilities of people living with dementia to control everyday decision-making preferences. The majority of the findings are however, about home based care identifying that care partners were chosen as primary substitute decision-makers, but financial strain was correlated with how well people living with dementia felt they were understood [24, 42, 49].

Decision-making participation characteristics were explored in a qualitative study of 21 care dyad interviews with older people living with dementia and their care partners [12]. Aspects explored were; information received, being listened to, ability to express an opinion, time allowed for reflection, and opportunity to change the decision. People living with dementia felt that they were not given enough time to reflect on decisions and did not feel their views about care provision were listened to. Care partners reported greater satisfaction with the quality of the communication and decision-making process [12].

Only one study explored the person living with dementia's *desire* to participate in decision-making [46]. Using a vignette, the study examined whether 48 people living with Alzheimer's disease would wish to participate in the decision to take a disease slowing medication, and what factors (including family) influenced their participation. In total 92% of people living with dementia wanted to participate in the decision, whilst only 71% of their care partners thought they would. People living with dementia concentrated on *involvement* in the *process*, whilst their care partners focused on their relatives' *capacity* to participate. Paradoxically, half of the people living with dementia that were formally assessed as lacking capacity (n=29) had care partners who said their relatives would be involved in decision-making [46].

For peer review only - http://bmjopen.bmj.com/site/about/guidelines.xhtml

Facilitators and inhibitors of shared decision-making

Care Partners and professionals

Where roles in decision-making are grounded in 'relationships' (or connectedness), the roles of care partners and workers can be facilitators or barriers [see e.g. 41, 44, 47, 52].

Only one study explicitly raised the question of who should participate in decision-making and what their roles should be [53]. Yet the impact of the relationship between the person living with dementia and their care partner on decision-making involvement is well documented [40, 46, 47, 49, 52]. There is little research focussing on shared decision-making relationships in extended care setting with the majority being undertaken in care dyads within the home environment [see e.g. 21, 22].

Clarke and colleagues [41] observed that care workers' characteristics could facilitate or inhibit decision-making involvement for people living with dementia in extended care settings. Positive characteristics included warmth, encouragement with memory, and routine. Routine also featured as a negative characteristic if linked to task orientated care. An example is given of a resident who appeared to be asleep and the carer had the person's feet out of bed before explaining what they intended to do [41 p20]. Other negative characteristics inhibiting shared decision-making were: discouraging independence, depersonalisation and risk adversity. The researchers observed more negative incidents than positive ones and felt that residents' autonomy was compromised [41]. Opportunities for expression of choice were reported to be reduced with the increase of daily care needs in extended care environments [45, 48]. Three studies identified reduced shared decision-making associated with: social attitudes, lack of available choices, systems, and care partners and workers identifying more problems than opportunities to involve the person living with dementia [11, 48, 53]. Optimal decision involvement was achieved by recognising the abilities and rights of the person living with a cognitive impairment as capable of influencing the decision, giving and sharing information, offering support and reinforcing opinions [11, 12, 40].

For peer review only - http://bmjopen.bmj.com/site/about/guidelines.xhtml

Tools and resources

Four studies developed and/or evaluated the use of tools and resources to facilitate shared decision-making for someone with cognitive impairment [39, 43, 51, 53]. Two studies evaluated the use of Talking Mats (TMs) which are a picture based, communication framework that allow people to indicate their feelings within a given topic by placing the relevant image on a visual scale. The first study compared TMs with structured and unstructured communication methods with five people in the late stages of Huntington's disease [43] and the second study compared TMs to usual communication methods with 18 people living with dementia and their care partners [51]. Both studies reported improvements in satisfaction with discussions when using TMs, although in one study [51] the feeling of involvement was significantly higher for the carers than for the people living with dementia.

Two studies developed computerised tools to support shared decision-making [39, 53]. Span and colleagues [53] co-designed an interactive web based tool to promote remote involvement of people living with dementia and their care partners in decision-making around such topics as; social contacts and daily activities, mobility and safety, future care, and finances [53]. Bailey and colleagues [39] created a visual aid for people living with a learning disability to facilitate participation in decision-making by presenting different types of information in a uniform format. They argue that this uniformity throughout a decision process could support everyday choices. The participants with a learning disability were trained to use the visual aid and their level of decision-making involvement improved as a result, although ongoing use was required to maintain the improvement [39].

Benefits of shared decision-making for people living with a cognitive impairment

Consulting residents living with dementia in an extended care environment, about the care home décor, Godwin [45] noted as an ancillary finding that residents appeared to be "surprised and pleased" to be asked [45 p. 114], arguing that this kind of consultation could enhance their self-esteem and contribute to their quality of life. Whilst this study did not measure the impact of the decision-making process, other studies have identified such benefits as heightened self-esteem, purpose and feeling of self-worth, as outcomes from retaining involvement in decision-making [44]. Ongoing decision-making involvement for

For peer review only - http://bmjopen.bmj.com/site/about/guidelines.xhtml

people living with dementia is also correlated with reduced depressive symptoms and maintained everyday functioning [24].

A rerun of the searches revealed three additional potentially relevant papers [57-59], however the Alzheimer's Society report [58] did not focus directly on shared decision-making. The qualitative study appraising how people living with dementia make decisions about daycare [57] confirmed the findings of the review in noting the crucial role that professionals can play in facilitating shared decisions. This work appears to be linked with an included study [53] and would suggest that while there is an ongoing interest in this topic intervention based work in care home settings remains limited. The study dedicated to shared decision-making in dementia care in care homes [59] is relevant however this paper reports only on the care staff perspective of the implementation of the study, and so does not meet the criteria for this review.

Discussion

The available evidence suggests that people living with cognitive impairment want the opportunity to participate in decision-making about their health and care; it can contribute to a sense of worth and has the potential to improve quality of life [12, 44, 46]. A lack of opportunity to participate in decision-making is a significant and consistent barrier throughout the literature reviewed [e.g. 11, 41, 48, 50]. This may be due to confusion about what shared decision-making is in everyday care for people living with cognitive impairment and whether it is in fact opportunities for greater choice that are required. Or it may be professionals' lack of skills in recognising and facilitating peoples' desire and ability to make a decision [50]. People living with dementia concentrated on *involvement* in the decision-making *process* [12, 44, 46] but there is a lack of evidence about how the person living with cognitive impairment chooses who they make decisions with and which resources or tools could facilitate people living with a cognitive impairment to *lead* the conversations.

Practical interventions to support and facilitate various aspects of the decision-making process (such as TMs and computer software tools) are reported as having good outcomes for the person living with a cognitive impairment, their care partner and in some cases their health or care professional too [39, 43, 51, 53]. However, current tools predominantly rely

For peer review only - http://bmjopen.bmj.com/site/about/guidelines.xhtml

on the care partner or professional to identify the decision topic, potentially disempowering the person living with a cognitive impairment. In addition implementing shared decision-making resources in extended care environments would require care workers being given the time, resource and authority to develop the skills required to use such aids. The included studies fail to provide evidence or discussion of the cost implications associated with embedding shared decision-making for people living with cognitive impairments, or the staff development needed to implement everyday decision-making in extended care settings.

Whether the importance of interdependent relationship between the (family) care partner and the person living with dementia in facilitating or inhibiting shared decision-making at home [40, 46, 47, 49, 52] is reflected by the relationship between the person living with dementia and their care staff in extended care settings is not yet understood. However, the frequent underestimation of care partners and workers of the desire and ability of people living with moderate and severe dementia to express preferences about their daily care [11, 12, 47, 48, 52] combined with the incongruence in levels of satisfaction in the decision-making process between care partners and people living with dementia [12, 46, 54] raises concerns about the role of consultees under the Mental Capacity Act (2005) [15] in the United Kingdom. Although as the process of decision-making may be as important as the decision itself [12, 42, 44, 45, 47], it could be argued that if all parties *perceive* that they have optimum levels of involvement and the desired outcomes are achieved the shared decision-making process is a success.

From the limited evidence available on how relationships in extended care affect shared decision-making it appears that how care staff engage is crucial. They can be enablers or blockers to shared decisions, and this appears to be related to their personality, communication skills and the routines of the workplace [41, 45, 50]. The implications of the relationships between people living with dementia in extended care and their care staff become increasingly significant as the dementia progresses with care needs increasing and communication capabilities dwindling. A greater understanding of the decision-making needs of the person living with dementia in extended care, and how they can be met, is therefore needed.

For peer review only - http://bmjopen.bmj.com/site/about/guidelines.xhtml

Strengths and Limitations

The review involved a systematic and rigorous search for literature relating to shared decision-making for people living with cognitive impairment in extended care settings. As such, this review provides a baseline to inform future research and practice. However, the majority of studies were conducted in the community rather than in extended care settings, quality was variable and there is little evidence on what supports the negotiation of day-to-day decisions between people with a cognitive impairment and their (staff and family) carers in extended care.

The review highlights the difficulties defining what is meant by shared decision-making for people who are cognitively impaired. It is recognised that terminology varies across Countries, disciplines and professions potentially impacting on the studies retrieved. Including additional search terms around 'choice' may have identified additional relevant papers that were not identified from terms relating to shared decision-making.

Quality assessment of qualitative studies was changed from the protocol [27] due to access issues so the QualSyst tool [35] was used. This tool has limitations particularly related to lack of requirement to assess an ethics statement.

Conclusion

Evidence suggests that people living with cognitive impairment value opportunities to be involved in everyday decision-making about their care although there remains some confusion about what constitutes shared decision-making in everyday care for people living with cognitive impairment in extended care. How to embed the process of shared decision-making into everyday practice in extended care and whether declining health and function are real or perceived barriers to participation remains to be determined along with the impact it would have on the quality of life of people living with dementia in care homes.

Further research is required to understand how opportunities for shared decision-making are created, recognised and understood; and whether they could improve the quality of life for people living with dementia in care homes. Research exploring the relationship between

For peer review only - http://bmjopen.bmj.com/site/about/guidelines.xhtml

the person living with dementia and their care staff would improve understanding of how shared decision-making can be better facilitated in extended care environments.

Patient and Public Involvement

The research question and systematic review objectives were presented to the members of the University of Hertfordshire Public Involvement in Research Group (UH PiRG), some of whom have experience of caring for family members with dementia. The group advised on the study design. The results of the review and the resulting study will be presented to the UH PiRG at one of their regular meetings.

Authors' contributions

RD wrote the protocol, undertook data extraction, quality assessment and analysis and wrote the paper. FB and CG supervised and critically appraised all aspects of the process. Important changes from the protocol have been explained. All authors read and approved the final manuscript. The authors are grateful to members of the UH PiRG for their input into the design of this review.

Funding statement

This review is undertaken as part of a wider doctoral study focusing on dementia care in care homes which has been funded by the National Institute for Health Research (NIHR) Collaboration for Leadership in Applied Health Research & Care (CLAHRC) East of England.

This report presents independent research funded by the National Institute for Health Research (NIHR) Collaboration for Leadership in Applied Health Research & Care (CLAHRC) East of England, at Cambridgeshire and Peterborough NHS Foundation Trust. The views expressed are those of the author(s) and not necessarily those of the NHS, the NIHR or the Department of Health.

Competing Interests

Authors declare that they have no competing interests.

Data Sharing Statement

No additional unpublished data are available for this review.

For peer review only - http://bmjopen.bmj.com/site/about/guidelines.xhtml

References

1. Edvardsson, D., D. Fetherstonhaugh, and R. Nay, *Promoting a continuation of self and normality: person-centred care as described by people with dementia, their family members and aged care staff.* Journal of clinical nursing, 2010. **19**(17-18): p. 2611-2618.

2. Hutchison, B., et al., *Primary health care in Canada: systems in motion.* Milbank Quarterly, 2011. **89**(2): p. 256-288.

3. Stiggelbout, A.M., et al., *Shared decision making: really putting patients at the centre of healthcare.* Bmj, 2012. **344**(S 28).

4. Wolfs, C.A., et al., *Rational decision-making about treatment and care in dementia: A contradiction in terms?* Patient Education & Counseling, 2012. **87**(1): p. 43-48 6p.

5. Edvardsson, D., B. Winblad, and P.-O. Sandman, *Person-centred care of people with severe Alzheimer's disease: current status and ways forward.* The Lancet Neurology, 2008. **7**(4): p. 362-367.

6. McCormack, B., *Person-centredness in gerontological nursing: an overview of the literature.* Journal of clinical nursing, 2004. **13**(s1): p. 31-38.

7. CQC, C.Q.C., *Care Homes.* 2009.

8. Bartlett, R. and D. O'Connor, *Broadening the dementia debate: Towards social citizenship*. 2010: Policy Press.

9. Wilkinson, H., *Empowerment and decision-making for people with dementia: the use of legal interventions in Scotland.* Aging Ment Health, 2001. **5**(4): p. 322-8.

10. Bekker, H.L., *Using decision-making theory to inform clinical practice.* Shared decision-making in health care: achieving evidence-based patient choice, 2009. **2**: p. 45-51.

11. Smebye, K.L., Kirkevold, M. & Engedal, K. , *How do persons with dementia participate in decision making related to health and daily care? a multi-case study.* BMC Health Serv Res, 2012. **12**: p. 241.

12. Tyrrell, J.G., N.: Myslinski, M., *Freedom of choice and decision-making in health and social care: views of older patients with early-stage dementia and their carers.* Dementia (14713012), 2006. **5**(4): p. 479-502 24p.

For peer review only - http://bmjopen.bmj.com/site/about/guidelines.xhtml

13. Nations, U., *Convention on the Rights of Persons with Disabilities (CRPD)*, U. Nations, Editor. 2006, Division for Social Policy and Development Disability: New York.

14. Manthorpe, J.S., K., *Changing practice: Adapting to the Mental Capacity Act 2005.* Social Care and Neurodisability, 2013. **4**(3): p. 124-133.

15. Affairs, D.o.C., *Mental Capacity Act 2005*, D.o.C. Affairs, Editor. 2005, HMSO London.

16. Affairs, D.o.C., *Mental Capacity Act 2005; code of practice.* . 2007, TSO: London.

17. Boyle, G., *The Mental Capacity Act 2005: promoting the citizenship of people with dementia?* Health & Social Care in the Community, 2008. **16**(5): p. 529-537 9p.

18. Manthorpe, J.S., K., *Mental capacity and dementia: A review. Part 2.* Journal of Dementia Care, 2013. **21**(1): p. 35-38.

19. Goodman, C., et al., *Preferences and priorities for ongoing and end-of-life care: A qualitative study of older people with dementia resident in care homes.* International journal of nursing studies, 2013. **50**(12): p. 1639-1647.

20. Mathie, E., et al., *An uncertain future: the unchanging views of care home residents about living and dying.* Palliative Medicine, 2012. **26**(5): p. 734-743.

21. Miller, L.M., C.J. Whitlatch, and K.S. Lyons, *Shared decision-making in dementia: A review of patient and family carer involvement.* Dementia, 2014.

22. Larsson, A.T. and J.H. Österholm, *How are decisions on care services for people with dementia made and experienced? A systematic review and qualitative synthesis of recent empirical findings.* International Psychogeriatrics, 2014. **26**(11): p. 1849-1862.

23. Taghizadeh Larsson, A.O., J. H., *How are decisions on care services for people with dementia made and experienced? A systematic review and qualitative synthesis of recent empirical findings.* Int Psychogeriatr, 2014. **26**(11): p. 1849-62.

24. Menne, H.L.W., C. J., *Decision-making involvement of individuals with dementia.* Gerontologist, 2007. **47**(6): p. 810-9.

25. Mariani, E., et al., *Shared decision-making on a 'life-and-care plan'in long-term care facilities: research protocol.* Nursing Open, 2016.

26. Orrell, M., et al., *The needs of people with dementia in care homes: the perspectives of users, staff and family caregivers.* International Psychogeriatrics, 2008. **20**(05): p. 941-951.

For peer review only - http://bmjopen.bmj.com/site/about/guidelines.xhtml

27. Daly, R., F. Bunn, and C. Goodman, *Shared decision-making for people living with dementia in extended care settings: protocol for a systematic review.* BMJ open, 2016. **6**(11): p. e012955.

28. Moher, D., et al., *Preferred reporting items for systematic review and meta-analysis protocols (PRISMA-P) 2015 statement.* Syst Rev, 2015. **4**: p. 1.

29. Braun, V. and V. Clarke, *Using thematic analysis in psychology.* Qualitative research in psychology, 2006. **3**(2): p. 77-101.

30. Kitwood, T., *The experience of dementia.* Aging & Mental Health, 1997. **1**(1): p. 13-22.

31. Kitwood, T., *The concept of personhood and its relevance for a new culture of dementia care*. 1997: London: Routledge.

32. Kitwood, T., *Dementia Reconsidered*. 1997, Buckingham, UK: Open University Press.

33. Laidsaar-Powell, R., et al., *Physician–patient–companion communication and decision-making: a systematic review of triadic medical consultations.* Patient Education and Counseling, 2013. **91**(1): p. 3-13.

34. Légaré, F., et al., *Barriers and facilitators to implementing shared decision-making in clinical practice: Update of a systematic review of health professionals' perceptions.* Patient Education and Counseling, 2008. **73**(3): p. 526-535.

35. Kmet L, L.R., Cook L., *Standard Quality Assessment Criteria for Evaluating Primary Research Papers from a Variety of Fields*. 2004, Alberta Heritage Foundation for Medical Research: Edmonton.

36. Noyes J, H.K., Booth A, Harris J, Harden A, Popay J, Pearson A, Cargo M, and Pantoja T, *Qualitative research and Cochrane reviews. In:*, in *Cochrane Handbook for Systematic Reviews of Interventions* G.S. Higgins JPT, Editor. 2015, The Cochrane Collaboration: http://qim.cochrane.org/supplemental-handbook-guidance.

37. Hannes, K., *Critical appraisal of qualitative research*, in *Supplementary Guidance for Inclusion of Qualitative Research in Cochrane Systematic Reviews of Interventions.*, B.A. Noyes J, Hannes K, Harden A, Harris J, Lewin S, Lockwood C. , Editor. 2011, Cochrane Collaboration Qualitative Methods Group: http://cqrmg.cochrane.org/supplemental-handbook-guidance.

38. Clarke, V. and V. Braun, *Thematic analysis*, in *Encyclopedia of critical psychology*. 2014, Springer. p. 1947-1952.

For peer review only - http://bmjopen.bmj.com/site/about/guidelines.xhtml

39. Bailey, R.W., P.: Dymond, S., *A visual aid to decision-making for people with intellectual disabilities.* Research in Developmental Disabilities, 2011. **32**(1): p. 37-46 10p.

40. Boyle, G., *Recognising the agency of people with dementia.* Disability & Society, 2014. **29**(7): p. 1130-1144 15p.

41. Clarke, A.M.D., M. F., *Communication and decision making among residents with dementia.* Geriaction, 2004. **22**(3): p. 17-24 8p.

42. Feinberg, L.F. and J. Whitlatch, *Decision-making for persons with cognitive impairment and their family caregivers.* American journal of Alzheimer's disease and other dementias, 2002. **17**(4): p. 237-244.

43. Ferm, U.S., A.: Sundin, L.: Hartelius, L., *Using Talking Mats to support communication in persons with Huntington's Disease.* International Journal of Language & Communication Disorders, 2010. **45**(5): p. 523-536 14p.

44. Fetherstonhaugh, D.T., L.: Nay, R., *Being central to decision making means I am still here!: the essence of decision making for people with dementia.* J Aging Stud, 2013. **27**(2): p. 143-50.

45. Godwin, B., *Colour consultation with dementia home residents and staff.* Quality in Ageing & Older Adults, 2014. **15**(2): p. 102-119 18p.

46. Hirschman, K.B.J., C. M.: James, B. D.: Xie, S. X.: Karlawish, J. H., *Do Alzheimer's disease patients want to participate in a treatment decision, and would their caregivers let them?* Gerontologist, 2005. **45**(3): p. 381-8.

47. Horton-Deutsch, S.T., Prudence: Evans, Rebecca, *Health care decision-making of persons with dementia.* Dementia, 2007. **6**(1): p. 105-120.

48. Kjellberg, A., *More or less independent.* Disability & Rehabilitation, 2002. **24**(16): p. 828-840 13p.

49. Menne, H.L.T., S. S.: Whitlatch, C. J.: Feinberg, L. F., *Decision-making involvement scale for individuals with dementia and family caregivers.* Am J Alzheimers Dis Other Demen, 2008. **23**(1): p. 23-9.

50. Milte, C.M.R., Julie: Davies, Owen: Whitehead, Craig: Masters, Stacey: Crotty, Maria, *Family meetings for older adults in intermediate care settings: the impact of patient cognitive impairment and other characteristics on shared decision making.* Health Expectations, 2015. **18**(5): p. 1030-1040.

For peer review only - http://bmjopen.bmj.com/site/about/guidelines.xhtml

51. Murphy, J.O., T., *The use of Talking Mats to support people with dementia and their carers to make decisions together.* Health Soc Care Community, 2013. **21**(2): p. 171-80.

52. Samsi, K. and J. Manthorpe, *Everyday decision-making in dementia: findings from a longitudinal interview study of people with dementia and family carers.* International Psychogeriatrics, 2013. **25**(6): p. 949-961 13p.

53. Span, M., *Developing an interactive web tool to facilitate shared decision-making in dementia care networks: a participatory journey*. 2016, Amsterdam: Vrije Universiteit.

54. Whitlatch, C.J.F., Lynn Friss Tucke, Shandra S., *Measuring the Values and Preferences for Everyday Care of Persons with Cognitive Impairment and Their Family Caregivers.* The Gerontologist, 2005. **45**(3): p. 370-380.

55. Elwyn, G., et al., *Investing in deliberation: a definition and classification of decision support interventions for people facing difficult health decisions.* Medical Decision Making, 2010. **30**(6): p. 701-711.

56. Thompson, A.G., *The meaning of patient involvement and participation in health care consultations: a taxonomy.* Social science & medicine, 2007. **64**(6): p. 1297-1310.

57. Groen-van de Ven, L., et al., *Involvement of people with dementia in making decisions about their lives: a qualitative study that appraises shared decision-making concerning daycare.* BMJ open, 2017. **7**(11): p. e018337.

58. Society, A.s., *Turning Up the Volume: unheard voices of people with dementia*. 2017, Alzheimer's Society: https://www.ipsos.com/sites/default/files/2017-05/dementia-alzheimers-society-may-2017.pdf. p. 44.

59. Mariani, E., et al., *Shared decision-making in dementia care planning: barriers and facilitators in two European countries.* Aging & mental health, 2017. **21**(1): p. 31-39.

For peer review only - http://bmjopen.bmj.com/site/about/guidelines.xhtml

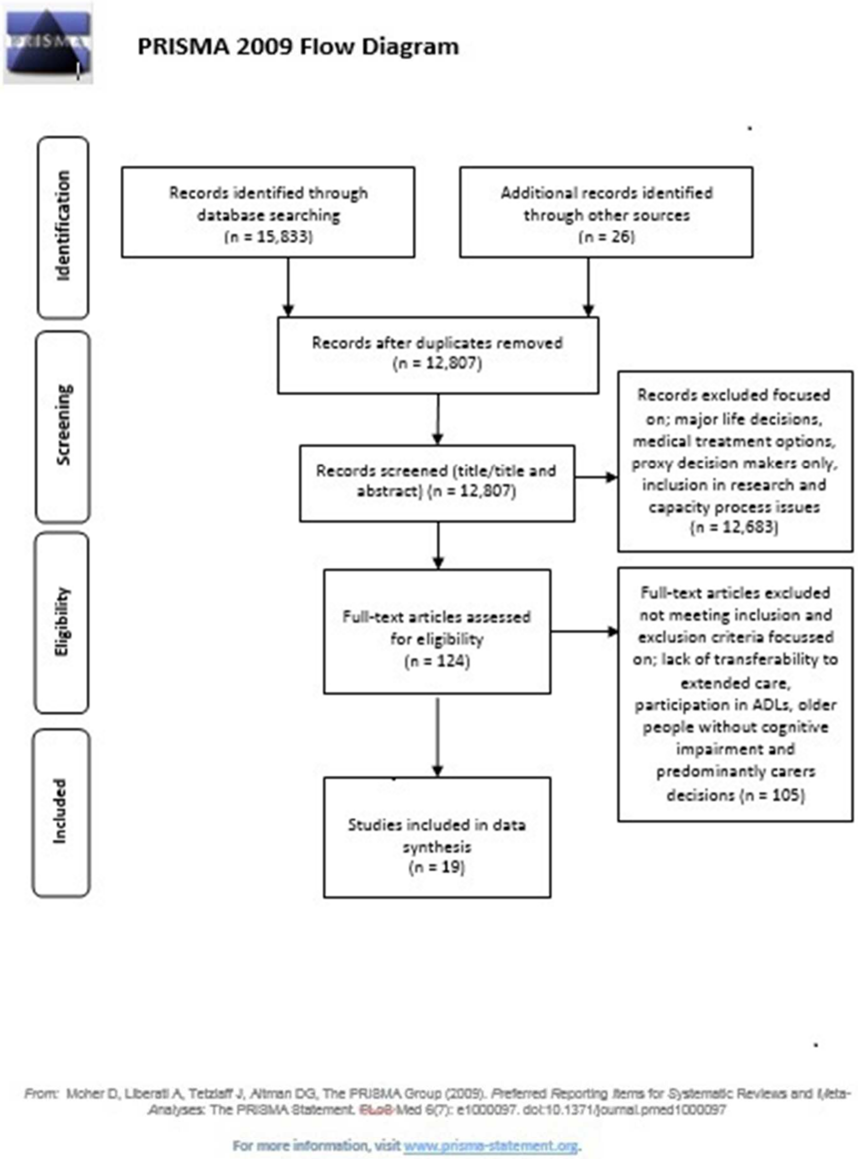

37x49mm (300 x 300 DPI)

PRISMA 2009 Checklist SHARED DECISION-MAKING FOR PEOPLE LIVING WITH DEMENTIA IN EXTENDED CARE SETTINGS: A SYSTEMATIC REVIEW

Section/topic	#	Checklist item	Reported on page #
**TITLE**			
Title	1	Identify the report as a systematic review, meta-analysis, or both.	3,4
**ABSTRACT**			
Structured summary	2	Provide a structured summary including, as applicable: background; objectives; data sources; study eligibility criteria, participants, and interventions; study appraisal and synthesis methods; results; limitations; conclusions and implications of key findings; systematic review registration number.	2
**INTRODUCTION**			
Rationale	3	Describe the rationale for the review in the context of what is already known.	3-4
Objectives	4	Provide an explicit statement of questions being addressed with reference to participants, interventions, comparisons, outcomes, and study design (PICOS).	4-5
**METHODS**			
Protocol and registration	5	Indicate if a review protocol exists, if and where it can be accessed (e.g., Web address), and, if available, provide registration information including registration number.	4
Eligibility criteria	6	Specify study characteristics (e.g., PICOS, length of follow-up) and report characteristics (e.g., years considered, language, publication status) used as criteria for eligibility, giving rationale.	5
Information sources	7	Describe all information sources (e.g., databases with dates of coverage, contact with study authors to identify additional studies) in the search and date last searched.	6-7
Search	8	Present full electronic search strategy for at least one database, including any limits used, such that it could be repeated.	6-7
Study selection	9	State the process for selecting studies (i.e., screening, eligibility, included in systematic review, and, if applicable, included in the meta-analysis).	8
Data collection process	10	Describe method of data extraction from reports (e.g., piloted forms, independently, in duplicate) and any processes for obtaining and confirming data from investigators.	8
Data items	11	List and define all variables for which data were sought (e.g., PICOS, funding sources) and any assumptions and simplifications made.	5
Risk of bias in individual studies	12	Describe methods used for assessing risk of bias of individual studies (including specification of whether this was done at the study or outcome level), and how this information is to be used in any data synthesis.	8
Summary measures	13	State the principal summary measures (e.g., risk ratio, difference in means).	N/A
Synthesis of results	14	Describe the methods of handling data and combining results of studies, if done, including measures of consistency (e.g., I^2) for each meta-analysis.	N/A

For peer review only

PRISMA 2009 Checklist SHARED DECISION-MAKING FOR PEOPLE LIVING WITH DEMENTIA IN EXTENDED CARE SETTINGS: A SYSTEMATIC REVIEW

Section/topic	#	Checklist item	Reported on page #
Risk of bias across studies	15	Specify any assessment of risk of bias that may affect the cumulative evidence (e.g., publication bias, selective reporting within studies).	8
Additional analyses	16	Describe methods of additional analyses (e.g., sensitivity or subgroup analyses, meta-regression), if done, indicating which were pre-specified.	N/A
**RESULTS**			
Study selection	17	Give numbers of studies screened, assessed for eligibility, and included in the review, with reasons for exclusions at each stage, ideally with a flow diagram.	8-10
Study characteristics	18	For each study, present characteristics for which data were extracted (e.g., study size, PICOS, follow-up period) and provide the citations.	10
Risk of bias within studies	19	Present data on risk of bias of each study and, if available, any outcome level assessment (see item 12).	9
Results of individual studies	20	For all outcomes considered (benefits or harms), present, for each study: (a) simple summary data for each intervention group (b) effect estimates and confidence intervals, ideally with a forest plot.	11-17
Synthesis of results	21	Present results of each meta-analysis done, including confidence intervals and measures of consistency.	N/A
Risk of bias across studies	22	Present results of any assessment of risk of bias across studies (see Item 15).	9
Additional analysis	23	Give results of additional analyses, if done (e.g., sensitivity or subgroup analyses, meta-regression [see Item 16]).	N/A
**DISCUSSION**			
Summary of evidence	24	Summarize the main findings including the strength of evidence for each main outcome; consider their relevance to key groups (e.g., healthcare providers, users, and policy makers).	17-18
Limitations	25	Discuss limitations at study and outcome level (e.g., risk of bias), and at review-level (e.g., incomplete retrieval of identified research, reporting bias).	18
Conclusions	26	Provide a general interpretation of the results in the context of other evidence, and implications for future research.	19
**FUNDING**			
Funding	27	Describe sources of funding for the systematic review and other support (e.g., supply of data); role of funders for the systematic review.	20

From: Moher D, Liberati A, Tetzlaff J, Altman DG, The PRISMA Group (2009). Preferred Reporting Items for Systematic Reviews and Meta-Analyses: The PRISMA Statement. PLoS Med 6(7): e1000097. doi:10.1371/journal.pmed1000097

For more information, visit: www.prisma-statement.org.

For peer review only - http://bmjopen.bmj.com/site/about/guidelines.xhtml

BMJ Open

SHARED DECISION-MAKING FOR PEOPLE LIVING WITH DEMENTIA IN EXTENDED CARE SETTINGS: A SYSTEMATIC REVIEW

Journal:	*BMJ Open*
Manuscript ID	bmjopen-2017-018977.R2
Article Type:	Research
Date Submitted by the Author:	15-Mar-2018
Complete List of Authors:	Daly, Rachel; University of Hertfordshire, CRIPACC<br>Bunn, Frances; University of Herfordshire, Department of Health and Human Sciences<br>Goodman, Claire; University of Hertfordshire, Centre for Research in Primary and Community Care
**Primary Subject Heading**:	Evidence based practice
Secondary Subject Heading:	Mental health, Communication, Geriatric medicine, Patient-centred medicine
Keywords:	Dementia < NEUROLOGY, Delirium & cognitive disorders < PSYCHIATRY, shared decision-making, Care Homes, Extended care, Everyday care

SCHOLARONE™
Manuscripts

For peer review only - http://bmjopen.bmj.com/site/about/guidelines.xhtml

Title: SHARED DECISION-MAKING FOR PEOPLE LIVING WITH DEMENTIA IN EXTENDED CARE SETTINGS: A SYSTEMATIC REVIEW.

Authors:

Rachel Daly, Queens Nurse, NIHR RDCP programme CLAHRC East of England doctoral student r.daly2@herts.ac.uk

Centre for Research in Primary and Community Care (CRIPACC), University of Hertfordshire, College Lane, Hatfield, Herts, England, AL10 9AB

Professor Frances Bunn, Professor in Health and Complex Conditions f.bunn@herts.ac.uk

Centre for Research in Primary and Community Care (CRIPACC), University of Hertfordshire, College Lane, Hatfield, Herts, England, AL10 9AB

Professor Claire Goodman, Professor Health Services Research c.goodman@herts.ac.uk

Centre for Research in Primary and Community Care (CRIPACC), University of Hertfordshire, College Lane, Hatfield, Herts, England, AL10 9AB

Corresponding author Rachel Daly – r.daly2@herts.ac.uk Tel: +44 7967 761298

Keywords: Cognitive Impairment, Dementia, Shared Decision-Making, Care Homes, Everyday Care, Extended Care, Day-to-Day Care, Residential Care

Tables – 3

References – 59

Supplementary files – Supplementary Figure 1 (Prisma flow chart)

For peer review only

For peer review only - http://bmjopen.bmj.com/site/about/guidelines.xhtml

ABSTRACT

Background – Shared decision-making is recognized as an important element of person-centered dementia care.

Objectives - The aim of this review was to explore how people living with dementia and cognitive impairment can be included in day-to-day decisions about their health and care in extended care settings

Design – A systematic review including primary research relating to shared decision-making, with cognitively impaired adults in (or transferrable to) extended care settings. Databases searched were: CINAHL, PubMed, the Cochrane Library, NICE Evidence, OpenGrey, Autism Data, Google Scholar, Scopus and Medicines Complete (June - October 2016 and updated 2018) for studies published in the last 20 years. PROSPERO number CRD42016035919

Results. Of the 19 included studies 15 involved people with living dementia, 7 in extended care settings. People living with cognitive impairment often have the desire and ability to participate in decision-making about their everyday care, although this is regularly underestimated by their staff and family care partners. Shared decision-making has the potential to improve quality of life for both the person living with dementia and those who support them. How resources to support shared decision-making are implemented in extended care settings, is less well understood.

Conclusions – Evidence suggests that people living with cognitive impairment value opportunities to be involved in everyday decision-making about their care. How these opportunities are created, understood, supported and sustained in extended care settings remains to be determined.

Strengths and limitations;

- The review involved a systematic and rigorous search for cross-disciplinary literature relating to shared decision-making for people living with cognitive impairment in extended care settings.
- The majority of studies were conducted in the community rather than in extended care settings.

For peer review only - http://bmjopen.bmj.com/site/about/guidelines.xhtml

- Terminology varies across Countries, disciplines and professions potentially impacting on the studies retrieved. Including additional search terms around 'choice' may have identified additional relevant papers.

Word Count – 4,571

SHARED DECISION-MAKING FOR PEOPLE LIVING WITH DEMENTIA IN EXTENDED CARE SETTINGS: A SYSTEMATIC REVIEW.

Background

For people living with dementia shared decision-making is increasingly considered crucial in health and care practice [1-4] and is an essential aspect of person centred care [5];the fundamental premise of which is that the individual's priorities, interests, abilities and character should inform decision-making [6]. Shared decision-making practice is equally important in extended care. Extended care is defined as residential settings that provide onsite care. This includes supported living, care villages and extra care housing in addition to more traditional care homes with and without nursing [7].

A dementia diagnosis does not automatically render someone incapable of making a decision [8, 9] but for a decision to truly be shared, mutuality must be established [10]. Legislative changes in the last decade have strengthened the rights of people living with dementia to participate in decisions about their care [11, 12]. The United Nations Convention on the Rights of People with Disabilities (CRPD), states that "persons with disabilities enjoy legal capacity on an equal basis with others in all aspects of life" [13]. However, evidence on *how* care decisions are currently made within various legislative frameworks, for and with, people living with dementia, is patchy and often focuses on decisions relating to life events [11, 14]. In England and Wales the Mental Capacity Act (2005) provides a legislative framework to protect and empower people to participate in decisions about their life and care unless there is evidence to the contrary [15, 16]. Evidence suggests however, that staff in extended care often make day-to-day care decisions on behalf of people living with dementia [17, 18]. There are situations where people living with dementia in extended care settings decline to participate in decision-making and prefer to relinquish control and delegate to a care partner or worker. Reasons identified include;

For peer review only - http://bmjopen.bmj.com/site/about/guidelines.xhtml

anxiety about ability to participate and reluctance to accept decreasing abilities (reported as self-protection) [see e.g. 19, 20, 21].

Two reviews have focused on shared decision-making with people living at home with dementia [21, 22]. They offer useful insight into degrees of involvement in the decision-making process, what influences that involvement and how people participate [21, 22].In a review of 36 studies of people living with dementia and their carers Miller [21] identified assorted patterns of decision-making ranging from 'being free to make a choice' through 'supported autonomy' to 'being reliant upon carers'. Larsson's review of 24 studies on community decisions relating to access to care services developed three themes to capture how people living with dementia experience involvement in care decisions; *excluded, prior preferences taken into account,* and *current preferences respected* [23]. These reviews usefully bring shared decision-making with people living with dementia into focus but largely excluded people living in extended care settings, who are typically frailer and further along the dementia trajectory. They highlight that decisions for people living with dementia at home invariably involved a care partner/family member. However, family members are often unavailable for many of the day-to-day decisions undertaken in extended care due to time and geographical constraints.

There is increasing recognition that day-to-day decisions are potentially more significant in everyday quality of life than the more noteworthy issues such as treatment decisions or relocation [see e.g. 24, 25, 26]. Building on previous work, this paper considers the evidence on *how* day-to-day decisions are understood and negotiated between people with a cognitive impairment and their (staff and family) carers in extended care settings. The review objectives are presented in *Box 1.*

**Box 1 – Review Objectives**
❖ Explore how shared decision making is understood and/or characterised for people living with dementia and their (staff and family) carers<br>❖ Explore the role of (staff and family) carers of people living with dementia in shared decision making care dyads<br>❖ Analyse identified risks and benefits associated with shared decision making for people with cognitive impairment<br>❖ Ascertain empirical evidence for the effectiveness of available shared decision making resources for people living with dementia<br>❖ Seek to understand the barriers and facilitators to effective shared decision making for people living

For peer review only - http://bmjopen.bmj.com/site/about/guidelines.xhtml

with dementia and their (staff and family) carers
- Explore the extent to which shared decision making has been researched in extended care settings
- Identify implications for shared decision making in dementia care practice, policy and future research

Methods

A detailed account to the review methods are published elsewhere [27] PROSPERO registration number CRD42016035919. The review was planned and is reported in accordance with the Preferred Reporting Items for Systematic Reviews and Meta-Analyses (PRISMA) guidelines [28].

Results are presented to reflect relevance to the review question and objectives rather than frequency that the topic arises in the data and must represent a pattern across more than one included study to constitute a theme [29].

Study selection and inclusion criteria

Inclusion criteria are presented in *Table 1* below. A time limit of 20 years was applied to reflect the rapidly changing literature on involving people living with dementia in their care, published since the seminal works of Tom Kitwood [30-32]. The main focus of the review was on people with dementia but studies relating to adults with other types of cognitive impairment were also included because of the potential for transferable learning.

**Table 1 - Inclusion Criteria**	
**Publication Language**	English
**Publication Dates**	January 1996 - October 2016
**Target Population**	Adults, aged over 18 years, with any type of cognitive impairment for example (but not limited to) dementia, learning disabilities, Parkinson's and Huntington's disease
**Study Setting**	Community living at home or extended care settings e.g. supported living, or residential care. Studies must be considered transferable to people living with a cognitive impairment in an extended care setting (for example the person living with a cognitive impairment must be in receipt of care in addition to that provided by a family care partner

For peer review only - http://bmjopen.bmj.com/site/about/guidelines.xhtml

	and the resource should be suitable for implementation in extended care settings.
**Study Types**	All research designs including quantitative and qualitative research.
**Primary Outcomes**	Any outcome relating to involvement in care planning, satisfaction with decision (e.g. care was congruent with expressed choice), quality of life or wellbeing, and behavioural changes (e.g. reduction in distress)
**Additional Outcomes**	Family care partner and/or health or care professional satisfaction, and any documented adverse effects (e.g. falls, weight loss, adverse outcomes related to medication management).

Table 1–study inclusion criteria

The term 'care partner' has been adopted to represent a family member or friend who has a caring relationship with the person living with a cognitive impairment. The terms health and/or care professionals, staff or workers are used interchangeably to describe people who are paid to provide health or social care.

Search strategy

The search strategy drew on a range of cross discipline data sources associated with cognitive impairment and dementia care. Electronic databases searched and an example of the search query for PubMed is given in *table 2*. Search terms were adapted as appropriate for other databases.

**Table 2 - Search Strategy**	
**Electronic databases searched**	CINAHL Plus, PubMed, the Cochrane Library, NICE Evidence, OpenGrey, Autism Data, Google Scholar, Scopus and MedicinesComplete
**MeSH search terms (with subheadings included)**	Cognition Disorders searched (((("dementia"[MeSH Terms] OR "dementia"[All Fields]) OR ("cognitive dysfunction"[MeSH Terms] OR ("cognitive"[All Fields] AND "dysfunction"[All Fields]) OR "cognitive dysfunction"[All Fields] OR ("cognitive"[All Fields] AND "impairment"[All Fields]) OR "cognitive impairment"[All Fields])) OR ("learning disorders"[MeSH Terms] OR ("learning"[All Fields] AND "disorders"[All Fields]) OR "learning disorders"[All Fields] OR ("learning"[All Fields] AND "disability"[All Fields]) OR "learning disability"[All Fields])) OR ("learning

For peer review only - http://bmjopen.bmj.com/site/about/guidelines.xhtml

	disorders"[MeSH Terms] OR ("learning"[All Fields] AND "disorders"[All Fields]) OR "learning disorders"[All Fields] OR ("learning"[All Fields] AND "disorder"[All Fields]) OR "learning disorder"[All Fields])) OR ("autistic disorder"[MeSH Terms] OR ("autistic"[All Fields] AND "disorder"[All Fields]) OR "autistic disorder"[All Fields] OR "autism"[All Fields])) OR ("stroke"[MeSH Terms] OR "stroke"[All Fields])) OR ("brain injuries"[MeSH Terms] OR ("brain"[All Fields] AND "injuries"[All Fields]) OR "brain injuries"[All Fields] OR ("brain"[All Fields] AND "injury"[All Fields]) OR "brain injury"[All Fields])) OR ("neurocognitive disorders"[MeSH Terms] OR ("neurocognitive"[All Fields] AND "disorders"[All Fields]) OR "neurocognitive disorders"[All Fields])) OR ("alzheimer disease"[MeSH Terms] OR ("alzheimer"[All Fields] AND "disease"[All Fields]) OR "alzheimer disease"[All Fields] OR "alzheimer"[All Fields])) AND Shared Decision-making (((((((shared[All Fields] AND ("Decision (Wash D C)"[Journal] OR "decision"[All Fields])) OR ("decision-making"[MeSH Terms] OR ("decision"[All Fields] AND "making"[All Fields]) OR "decision-making"[All Fields])) OR ("community participation"[MeSH Terms] OR ("community"[All Fields] AND "participation"[All Fields]) OR "community participation"[All Fields] OR ("consumer"[All Fields] AND "participation"[All Fields]) OR "consumer participation"[All Fields])) OR ("patient participation"[MeSH Terms] OR ("patient"[All Fields] AND "participation"[All Fields]) OR "patient participation"[All Fields])) OR (("Decision (Wash D C)"[Journal] OR "decision"[All Fields]) AND support[All Fields])) OR (care[All Fields] AND dyad[All Fields])) OR ("patient education handout"[Publication Type] OR "patient education as topic"[MeSH Terms] OR "patient education"[All Fields]))) NOT (((advance[All Fields] AND ("Decision (Wash D C)"[Journal] OR "decision"[All Fields])) OR ("advance directives"[MeSH Terms] OR ("advance"[All Fields] AND "directives"[All Fields]) OR "advance directives"[All Fields] OR ("advance"[All Fields] AND "directive"[All Fields]) OR "advance directive"[All Fields])) OR (advance[All Fields] AND care[All Fields] AND plan[All Fields]))) OR (((((("Dementia"[Mesh] OR "Neurocognitive Disorders"[Mesh]) OR "Brain Injuries"[Mesh]) OR "Stroke"[Mesh]) OR "Learning Disorders"[Mesh]) OR "Autistic Disorder"[Mesh]) AND ((((("Decision-making"[Mesh] OR "Decision Support Techniques"[Mesh]) AND "Patient Participation"[Mesh]) OR "Cooperative Behavior"[Mesh]) OR "Physician-Patient Relations"[Mesh]) OR "Patient Education as Topic"[Mesh])) NOT Advance Directives("Advance Directives"[Mesh] OR "Advance Care Planning"[Mesh]))) NOT Paediatrics ("child"[MeSH Terms] OR "child"[All Fields])) NOT ("Child"[Mesh] OR "Disabled Children"[Mesh])
**Alternate free text search terms**	(Cogniti*, Disorder*, Dementia*, Alzheimer*, Neurocogniti* Dis*, Brain Injur*, Autis*, Learning Dis*, Stroke) AND (Shared Decision-making, Deci* Mak*, Patient Participat*, Consumer Participat* , Cooperat*, Decision Support) NOT (Paed* Child*) NOT (Advance Directives, Advance* care planning, Advance* deci*)
**Google Scholar**	The search and screening strategy for Google Scholar was agreed by all 3 authors. Free text search terms mirrored other databases. Results were filtered by relevance. The first 20 pages of results, title and abstract, were screened (20 results per page).

Table 2- Electronic databases searched, and search terms used

For peer review only - http://bmjopen.bmj.com/site/about/guidelines.xhtml

Reference lists of relevant primary and review articles were manually searched to detect additional studies and citation search was performed using the 'cited by' option on Google Scholar, and the 'related articles' option in PubMed. Searches were initially undertaken in June 2016 and updated in October 2016. Lateral searches were completed in November 2016. The search was updated in January 2018.

Electronic search results were downloaded into EndNote. One author (RD) screened references and, where necessary, sought support and independent review from a second author (FB or CG).

Quality Assessment and Data Extraction

Quality assessment was undertaken using the validated QualSyst Framework [33-35]. The QualSyst framework provides comprehensive definitions, instructions, and a scoring system for quality scoring of both quantitative and qualitative studies represented as a percentage (with a greater percentage representing higher quality)[35]. An ethical approval question was added as this has been highlighted as an essential element of study quality [36, 37]. Quality assessment was carried out by one reviewer (RD), with 10% checked by a second reviewer (FB/CG).

Data were extracted using a structured form that addressed the review objectives. This included information about the study design, participants and outcomes.

Analysis

Due to heterogeneity, and low numbers, of included quantitative studies, meta-analysis was not considered appropriate therefore results are reported in a narrative format.

Theoretical thematic analysis was undertaken using the research question and review objectives (see *box 1*) as a framework to map the range of data and identify recurrent themes. This method of synthesising qualitative research draws on work by Braun and Clarke [29, 38]. It offers in depth exploration of the identified themes and areas of interest,

For peer review only - http://bmjopen.bmj.com/site/about/guidelines.xhtml

which include the roles, resources, and people essential to the shared decision-making process relevant to extended care settings.

Results

Nineteen publications are included [11, 12, 24, 39-54](see *table 3*) relating to 18 unique studies (including a doctoral dissertation). Four of the papers are linked as they included a baseline sample drawn from the same pool of participants [24, 42, 49, 54]. However, the studies addressed different questions and are, therefore, included as individual papers. An overview of the screening and selection process is demonstrated in supplementary Figure 1.

Supplementary Figure 1 - PRISMA flow chart

Study Quality

Study quality ranged from 65-100% see *table 3*. No studies were excluded as a result of the quality assessment but a high risk of bias was noted in some studies, such as a measure designed by the same people as the intervention [43]. Eight studies did not provide a clear ethics statement [12, 24, 41-44, 51, 54], including the two studies that scored 100% on QualSyst [49, 54].

Characteristics of included studies and participants

All included studies (see *table 3*) were published between 2002 and 2016. The majority (n=14) of studies were published in the last 10 years, suggesting an increasing awareness and interest in shared decision-making for people living with a cognitive impairment that reflects the progression in national and international legislation. All five UK studies were published since the full implementation of the Mental Capacity Act [15].

Most papers (n=15) focus on people living with dementia [11, 12, 24, 40-42, 44-47, 49, 51-54]; twelve are in 'care dyads' (with a care partner) or 'triads' (with a care partner and a health or care worker). Two papers centre on people with a learning disability [39, 48], one on people living with Huntington's disease [43] , and one includes some people with cognitive

For peer review only - http://bmjopen.bmj.com/site/about/guidelines.xhtml

impairment [50]. Seven studies represent some participants living in extended care settings [11, 12, 41, 45, 46, 48, 50, 53], whilst the majority (n=12) relate to people living at home. Of the 19 studies included, only two studies specified *shared decision-making in extended care settings* as their explicit focus [41, 45].

The majority of studies (n=15) employed interviews and/or observations (see *table 3*). Three of the linked studies used structured interviews and compared the views of people living with a cognitive impairment and their care partners [42, 49, 54]. One study, used structured observations of family meetings [50]. Three studies undertook ethnographic observations of people living with dementia within a care dyad or triad [11, 40, 41]. Four studies identified person centred care as a theoretical framework [40, 45, 51, 53].

A breakdown of whether studies aimed to understand, evaluate interventions or measure shared decision-making is presented in *table 3* and discussed within the narrative below. Results are presented in cross-cutting themes that explore decision-making participation or involvement for people living with a cognitive impairment in terms of; how shared decision-making is understood and how participation in decision-making is measured, facilitated and inhibited.
Results are presented to reflect relevance to the review question and objectives rather than frequency that the topic arises in the data [29].

For peer review only

For peer review only - http://bmjopen.bmj.com/site/about/guidelines.xhtml

Table 3 - Characteristics of included studies

Author(s) (year) and [reference No.]	Purpose of study	Country	Methods	Quality score	Setting	Participants	Sample size: PLWCI	CP	CW	Total	Results or Outcomes
**Clarke, A. M. (2004) [41]**	To influence SDM	Australia 3	Qualitative Interviews and observations	75%	extended care	PLWD and CW	13		13	26	4 positive and 5 negative carer characteristics were identified that impacted on decision-making.
**Fetherston-haugh, D. (2013) [44]**	To understand SDM		Qualitative Interviews	80%	home	PLWD		6		6	3 pairs of conflicting attributes identified: 1) subtle support versus taking over; 2) hanging on versus letting go; and 3) being central versus being excluded
**Milte, C. (2015) [50]**	To understand SDM		Quantitative Observed family meetings	83%	intermediate care	Older people, CPs and HCPs	51	51	2	104	Geriatricians' performance in SDM was mixed; above baseline skill level in some areas and below in others. Longer meetings = better SDM by clinicians.
**Tyrrell, J. (2006) [12]**	To measure SDM	France 1	Qualitative Interviews	83%	home	PLWD and care partner	21	21		42	PLWD did not feel listened to and had limited freedom to participate in decision-making. Carers were more satisfied than PLWD
**Span, M. (2016) [53]**	To facilitate SDM	Holland 1	Qualitative Interviews, focus groups, specialist consultation and workshops	75%	home	PLWD, CPs and HCPs				84	18 topics of problems and 8 topics addressing decision-making in emerged. Only 8 topics were identified by both PLWD and Care partners
**Smebye, K. L. (2012) [11]**	To understand SDM	Norway 1	Mixed methods Interviews and observations	95%	home and extended care	PLWD, CPs and HCPs	10	10	10	30	Care staff do not base mental competence on standardized tests; values and relationships as important as logic. New decision-making categories emerged. Autonomous decision-making occurred but SDM was most typical.
**Ferm, U. (2010) [43]**	To facilitate SDM	Sweden 2	Mixed methods Interviews	95%	home	PLW HD	5			5	Talking Mats increased communication but effectiveness depended on conversation topic.

For peer review only

**Kjellberg, A. (2002) [48]**	To understand		Qualitative Interviews	70%	home, extended and day care	People living with LD	23			23	Of the 9 theoretical combinations of levels of decision-making identified only 5 emerged.
**Bailey, R. (2011) [39]**	To measure SDM	United Kingdom 5	Quantitative Electronic decision-making tasks and questionnaire	79%	Day services	People living with LD	24			24	Decision-making task performances improved when using the visual aid designed. Although not sustained without the visual aid the improvement was regained when the aid and was reintroduced.
**Boyle, G. (2014) [40]**	To measure SDM		Qualitative Creative interaction, observation and interviews	85%	home	PLWD and CPs	5	5		10	Identified that agency related to SDM is demonstrated within six relevant themes
**Godwin, B. (2014) [45]**	To facilitate SDM		Mixed methods Consultation	90%	extended care	PLWD	34		42	76	Residents were able to demonstrate preferences relating to their environment and enjoyed the consultation process.
**Murphy, J. (2013) [51]**	To facilitate SDM		Mixed methods Researcher facilitated discussion	65%	home	PLWD and CPs	18	18		18	Participants felt more involved in discussions when using Talking Mats although feeling of involvement was significantly higher for carers than for PWLD
**Samsi, K. (2013) [52]**	To understand SDM		Qualitative Interviews	90%	home	PLWD and CPs	15	15		30	3 underlying principles identified of decision-making is negotiated and how dynamics changed: Importance of autonomy, decision specific approach and made on someone's behalf described as' best interest'.
**Feinberg, L. F. (2002) [42]**	To measure SDM	United States of America 6	Quantitative Interviews	80%	home	PLWD and CPs	51	51		102	Lower income and carer financial strain correlated with how well the PLWD felt their carer knew their care wishes (more financial strain = less understanding)
**Hirschman, K. B. (2005)[46]**	To understand SDM		Interviews	70%	Home and extended care	PLWD and CPs	48	48		96	Spousal carer - wife (90%) vs husband (21%). Half care partners of PLWD formally 'lacking

For peer review only - http://bmjopen.bmj.com/site/about/guidelines.xhtml

											capacity' still involved them in decision-making.
**Horton-Deutsch, S. (2007) [47]**	To understand SDM		Mixed methods Interviews	85%	home	PLWD and CPs	20	20		40	75% PLWD had always involved HCP and/or spouse in decisions. 50% PLWD decisions changed 80% in line with CP wishes. Only 55% of dyads congruent throughout. 20% PLWD maintained choice.
**Menne, H. L. (2008) [49]**	To measure SDM		Quantitative Interviews Demographic information inc. MMSE scores Capacity evaluation	100 %	home	PLWD and CPs	217	217		434	PLWD consistently considered themselves to have more involvement in decision-making than their care partners perceived them to be
**Menne, H. L. (2007) [24]**	To measure SDM		Quantitative Secondary data analysis	86%	home	PLWD and CPs	215	215		430	Greater decision-making involvement associated with younger, female, educated, non-spousal CP, fewer months since diagnosis, fewer problems with ADLs, fewer depressive symptoms, and place more importance on autonomy/self-identity
**Whitlatch, C. (2005) [54]**	To measure SDM		Mixed methods Interviews	100 %	home	PLWD and CPs	111	111		222	Values and preferences correlated with CP perceptions of PLWD quality of life and involvement in decision-making and with PLWD perception of own quality of life and involvement in decision-making.

HD – Huntington's Disease, SDM – shared decision-making, PLWCI – person living with cognitive impairment, PLWD – person living with dementia, LD – Learning disability CP – care partner, CW –care worker, HCP – health care professional

Table 3 - Characteristics of included studies

For peer review only

For peer review only - http://bmjopen.bmj.com/site/about/guidelines.xhtml

Understanding 'shared decision-making' for people living with cognitive impairment

There was no common understanding of what shared decision-making entails and how it can be operationalised for people living with a cognitive impairment. Only one paper offered a definition of shared decision-making. Defining it as an approach that involves patients in making medical decisions with their clinician [55], is influenced by the type and complexity of the decision being made and the perspectives of care partners as well as the person living with cognitive impairment.

Three ways that people living with dementia understand shared decision-making were identified in a phenomenological study [44]; subtle support versus taking over; hanging on versus letting go; and being central versus being marginalised or excluded. One participant described their negative response to being excluded;

> *"…if someone came in and started telling me how I should run things or do things, I think I would certainly retaliate and not conform to anything they would want to do."* [44 p.147].

A recurrent theme in the qualitative literature reviewed is that for many people living with dementia, it is the participation or 'sharing' in the decision-making process, that is as, (if not more) important than making the decision itself [12, 42, 44, 47]. Despite this Samsi and Manthorpe [52] identified examples of 'substituted decision-making' that essentially excluded the person living with dementia;

> *"Oh I don't ask her what she wants anymore. I know what she'll say anyway - 'anything you like, you decide', so I just do what's best for us both."* [52 p958]

Participation in decision-making

The extent to which a person living with dementia is able to participate in decision-making was a focus of many of the included studies. For the purpose of this paper participation in decision-making is subdivided into the degree or level of participation, ability to participate and desire to participate.

For peer review only - http://bmjopen.bmj.com/site/about/guidelines.xhtml

Levels of participation in decision-making

Five studies addressed levels of participation [11, 12, 47, 48, 52]. Smebye and colleagues used Thompson's five point taxonomy of patient involvement and participation in health care consultations [56]. Thompsons taxonomy ranges from entirely passive 'non-involvement', through cooperative 'shared decision-making', to independent 'autonomous decision-making' [56]. Smebye identified two additional elements through care triad observations and interviews; pseudo-autonomous, (assumptions about a person's decision) and delegated (the person living with dementia expressly delegates their decision-making) when including people living with dementia in care triads [11]. This extended taxonomy is reflected in all five studies exploring the extent of decision-making participation.

Shared-decision-making was considered as the most common decision-making pattern by people living with a cognitive impairment. Horton-Deutsch and colleagues [47] explored self-reported participation in decision-making for 20 people living with dementia and their care partners' using semi-structured interviews and a five point decision making scale centred on a treatment vignette. The scale ranged from (1) made decisions alone in the past with little input from others and continue to do so, to (5) discussed decisions with spouse in the past and continues to do so. Participants reported that although their self-perceived decision-making changed over time from, largely independent historically towards current interdependent (shared) decision-making, the majority (75%) of participants described some level of shared decision-making throughout [47]. Similarly, in a group of 23 people living with varying levels of learning disability, 70% rarely considered themselves entirely independent and relied on shared decision-making in some or all areas of their life [48].

Ability to participate

Seven studies [17, 39, 41, 42, 46, 49, 54] explored individuals' ability to participate in decisions often in the context of facilitators or barriers [e.g. 41]. All the studies presenting measures of decision-making participation compared the responses of the person living with a cognitive impairment with their care partners. They highlighted consistent incongruence between responses, with care partners typically believing the person living with dementia to be less involved than they perceived themselves [46, 54].

For peer review only - http://bmjopen.bmj.com/site/about/guidelines.xhtml

Five papers describe and/or evaluate tools that measure aspects of decision-making participation from the perspective of the person living with a cognitive impairment [12, 42, 44, 45, 47]. The linked studies (with an overlapping sample ranging from 51-217 care dyads) develop, use and evaluate the Decision Control Inventory (DCI) and the Decision-Making Involvement Scale (DMI) to assess and compare everyday care choices of both the person living with a cognitive impairment and their care partner [24, 42, 49, 54]. The DMI Scale measures involvement in everyday decisions such as what food to eat and when to go to bed. It aims to increase participation through communication and improve care planning [54]. The DCI explores the abilities of people living with dementia to control everyday decision-making preferences. The majority of the findings are however, about home based care identifying that care partners were chosen as primary substitute decision-makers, but financial strain was correlated with how well people living with dementia felt they were understood [24, 42, 49].

Decision-making participation characteristics were explored in a qualitative study of 21 care dyad interviews with older people living with dementia and their care partners [12]. Aspects explored were; information received, being listened to, ability to express an opinion, time allowed for reflection, and opportunity to change the decision. People living with dementia felt that they were not given enough time to reflect on decisions and did not feel their views about care provision were listened to. Care partners reported greater satisfaction with the quality of the communication and decision-making process [12].

Only one study explored the person living with dementia's *desire* to participate in decision-making [46]. Using a vignette, the study examined whether 48 people living with Alzheimer's disease would wish to participate in the decision to take a disease slowing medication, and what factors (including family) influenced their participation. In total 92% of people living with dementia wanted to participate in the decision, whilst only 71% of their care partners thought they would. People living with dementia concentrated on *involvement* in the *process*, whilst their care partners focused on their relatives' *capacity* to participate. Paradoxically, half of the people living with dementia that were formally assessed as lacking capacity (n=29) had care partners who said their relatives would be involved in decision-making [46].

For peer review only - http://bmjopen.bmj.com/site/about/guidelines.xhtml

Facilitators and inhibitors of shared decision-making

Care Partners and professionals

Where roles in decision-making are grounded in 'relationships' (or connectedness), the roles of care partners and workers can be facilitators or barriers [see e.g. 41, 44, 47, 52].

Only one study explicitly raised the question of who should participate in decision-making and what their roles should be [53]. Yet the impact of the relationship between the person living with dementia and their care partner on decision-making involvement is well documented [40, 46, 47, 49, 52]. There is little research focussing on shared decision-making relationships in extended care setting with the majority being undertaken in care dyads within the home environment [see e.g. 21, 22].

Clarke and colleagues [41] observed that care workers' characteristics could facilitate or inhibit decision-making involvement for people living with dementia in extended care settings. Positive characteristics included warmth, encouragement with memory, and routine. Routine also featured as a negative characteristic if linked to task orientated care. An example is given of a resident who appeared to be asleep and the carer had the person's feet out of bed before explaining what they intended to do [41 p20]. Other negative characteristics inhibiting shared decision-making were: discouraging independence, depersonalisation and risk adversity. The researchers observed more negative incidents than positive ones and felt that residents' autonomy was compromised [41]. Opportunities for expression of choice were reported to be reduced with the increase of daily care needs in extended care environments [45, 48]. Three studies identified reduced shared decision-making associated with: social attitudes, lack of available choices, systems, and care partners and workers identifying more problems than opportunities to involve the person living with dementia [11, 48, 53]. Optimal decision involvement was achieved by recognising the abilities and rights of the person living with a cognitive impairment as capable of influencing the decision, giving and sharing information, offering support and reinforcing opinions [11, 12, 40].

For peer review only - http://bmjopen.bmj.com/site/about/guidelines.xhtml

Tools and resources

Four studies developed and/or evaluated the use of tools and resources to facilitate shared decision-making for someone with cognitive impairment [39, 43, 51, 53]. Two studies evaluated the use of Talking Mats (TMs) which are a picture based, communication framework that allow people to indicate their feelings within a given topic by placing the relevant image on a visual scale. The first study compared TMs with structured and unstructured communication methods with five people in the late stages of Huntington's disease [43] and the second study compared TMs to usual communication methods with 18 people living with dementia and their care partners [51]. Both studies reported improvements in satisfaction with discussions when using TMs, although in one study [51] the feeling of involvement was significantly higher for the carers than for the people living with dementia.

Two studies developed computerised tools to support shared decision-making [39, 53]. Span and colleagues [53] co-designed an interactive web based tool to promote remote involvement of people living with dementia and their care partners in decision-making around such topics as; social contacts and daily activities, mobility and safety, future care, and finances [53]. Bailey and colleagues [39] created a visual aid for people living with a learning disability to facilitate participation in decision-making by presenting different types of information in a uniform format. They argue that this uniformity throughout a decision process could support everyday choices. The participants with a learning disability were trained to use the visual aid and their level of decision-making involvement improved as a result, although ongoing use was required to maintain the improvement [39].

Benefits of shared decision-making for people living with a cognitive impairment

Consulting residents living with dementia in an extended care environment, about the care home décor, Godwin [45] noted as an ancillary finding that residents appeared to be "surprised and pleased" to be asked [45 p. 114], arguing that this kind of consultation could enhance their self-esteem and contribute to their quality of life. Whilst this study did not measure the impact of the decision-making process, other studies have identified such benefits as heightened self-esteem, purpose and feeling of self-worth, as outcomes from retaining involvement in decision-making [44]. Ongoing decision-making involvement for

For peer review only - http://bmjopen.bmj.com/site/about/guidelines.xhtml

people living with dementia is also correlated with reduced depressive symptoms and maintained everyday functioning [24].

A rerun of the searches revealed three additional potentially relevant papers [57-59], however the Alzheimer's Society report [58] did not focus directly on shared decision-making. The qualitative study appraising how people living with dementia make decisions about daycare [57] confirmed the findings of the review in noting the crucial role that professionals can play in facilitating shared decisions. This work appears to be linked with an included study [53] and would suggest that while there is an ongoing interest in this topic intervention based work in care home settings remains limited. The study dedicated to shared decision-making in dementia care in care homes [59] is relevant however this paper reports only on the care staff perspective of the implementation of the study, and so does not meet the criteria for this review.

Discussion

The available evidence suggests that people living with cognitive impairment want the opportunity to participate in decision-making about their health and care; it can contribute to a sense of worth and has the potential to improve quality of life [12, 44, 46]. A lack of opportunity to participate in decision-making is a significant and consistent barrier throughout the literature reviewed [e.g. 11, 41, 48, 50]. This may be due to confusion about what shared decision-making is in everyday care for people living with cognitive impairment and whether it is in fact opportunities for greater choice that are required. Or it may be professionals' lack of skills in recognising and facilitating peoples' desire and ability to make a decision [50]. People living with dementia concentrated on *involvement* in the decision-making *process* [12, 44, 46] but there is a lack of evidence about how the person living with cognitive impairment chooses who they make decisions with and which resources or tools could facilitate people living with a cognitive impairment to *lead* the conversations.

Practical interventions to support and facilitate various aspects of the decision-making process (such as TMs and computer software tools) are reported as having good outcomes for the person living with a cognitive impairment, their care partner and in some cases their health or care professional too [39, 43, 51, 53]. However, current tools predominantly rely

For peer review only - http://bmjopen.bmj.com/site/about/guidelines.xhtml

on the care partner or professional to identify the decision topic, potentially disempowering the person living with a cognitive impairment. In addition implementing shared decision-making resources in extended care environments would require care workers being given the time, resource and authority to develop the skills required to use such aids. The included studies fail to provide evidence or discussion of the cost implications associated with embedding shared decision-making for people living with cognitive impairments, or the staff development needed to implement everyday decision-making in extended care settings.

Whether the importance of interdependent relationship between the (family) care partner and the person living with dementia in facilitating or inhibiting shared decision-making at home [40, 46, 47, 49, 52] is reflected by the relationship between the person living with dementia and their care staff in extended care settings is not yet understood. However, the frequent underestimation of care partners and workers of the desire and ability of people living with moderate and severe dementia to express preferences about their daily care [11, 12, 47, 48, 52] combined with the incongruence in levels of satisfaction in the decision-making process between care partners and people living with dementia [12, 46, 54] raises concerns about the role of consultees under the Mental Capacity Act (2005) [15] in the United Kingdom. Although as the process of decision-making may be as important as the decision itself [12, 42, 44, 45, 47], it could be argued that if all parties *perceive* that they have optimum levels of involvement and the desired outcomes are achieved the shared decision-making process is a success.

From the limited evidence available on how relationships in extended care affect shared decision-making it appears that how care staff engage is crucial. They can be enablers or blockers to shared decisions, and this appears to be related to their personality, communication skills and the routines of the workplace [41, 45, 50]. The implications of the relationships between people living with dementia in extended care and their care staff become increasingly significant as the dementia progresses with care needs increasing and communication capabilities dwindling. A greater understanding of the decision-making needs of the person living with dementia in extended care, and how they can be met, is therefore needed.

For peer review only - http://bmjopen.bmj.com/site/about/guidelines.xhtml

Strengths and Limitations

The review involved a systematic and rigorous search for literature relating to shared decision-making for people living with cognitive impairment in extended care settings. As such, this review provides a baseline to inform future research and practice. However, the majority of studies were conducted in the community rather than in extended care settings, quality was variable and there is little evidence on what supports the negotiation of day-to-day decisions between people with a cognitive impairment and their (staff and family) carers in extended care.

The review highlights the difficulties defining what is meant by shared decision-making for people who are cognitively impaired. It is recognised that terminology varies across Countries, disciplines and professions potentially impacting on the studies retrieved. Including additional search terms around 'choice' may have identified additional relevant papers that were not identified from terms relating to shared decision-making.

Quality assessment of qualitative studies was changed from the protocol [27] due to access issues so the QualSyst tool [35] was used. This tool has limitations particularly related to lack of requirement to assess an ethics statement.

Conclusion

What constitutes shared decision-making in everyday care for people living with cognitive impairment in extended care remains unclear, which in turn leads to confusion about how to embed the process of shared decision-making into everyday practice in extended care. The significance of the interdependent relationships between people living with dementia in extended care and their care staff develops as dementia, care needs and communication difficulties increase. But whether declining health and function are real or perceived barriers to decision participation remains to be determined, along with the impact everyday shared decision-making would have on the quality of life of people living with dementia in care homes.

For peer review only - http://bmjopen.bmj.com/site/about/guidelines.xhtml

People living with cognitive impairment value opportunities to be *involved* in everyday decision-making about their care and involvement in the decision making process appears to be as important as the decision itself. This desire to share in decision-making is consistently underestimated by care partners and workers, which could have implications for the application of the Mental Capacity Act (2005) in practice. Tools and resources are shown to have a positive impact on decision-making participation, however in most instances, they do not empower the person living with a cognitive impairment to lead the decision.

Further research is required to understand how opportunities for shared decision-making are created, recognised and understood; and whether they could improve the quality of life for people living with dementia in care homes. Research exploring the relationship between the person living with dementia and their care staff would improve understanding of how shared decision-making can be better facilitated in extended care environments.

Patient and Public Involvement

The research question and systematic review objectives were presented to the members of the University of Hertfordshire Public Involvement in Research Group (UH PiRG), some of whom have experience of caring for family members with dementia. The group advised on the study design. The results of the review and the resulting study will be presented to the UH PiRG at one of their regular meetings.

Authors' contributions

RD wrote the protocol, undertook data extraction, quality assessment and analysis and wrote the paper. FB and CG supervised and critically appraised all aspects of the process. Important changes from the protocol have been explained. All authors read and approved the final manuscript. The authors are grateful to members of the UH PiRG for their input into the design of this review.

For peer review only - http://bmjopen.bmj.com/site/about/guidelines.xhtml

For peer review only

Funding statement

This review is undertaken as part of a wider doctoral study focusing on dementia care in care homes which has been funded by the National Institute for Health Research (NIHR) Collaboration for Leadership in Applied Health Research & Care (CLAHRC) East of England.

This report presents independent research funded by the National Institute for Health Research (NIHR) Collaboration for Leadership in Applied Health Research & Care (CLAHRC) East of England, at Cambridgeshire and Peterborough NHS Foundation Trust. The views expressed are those of the author(s) and not necessarily those of the NHS, the NIHR or the Department of Health.

Competing Interests

Authors declare that they have no competing interests.

Data Sharing Statement

No additional unpublished data are available for this review.

References

1. Edvardsson, D., D. Fetherstonhaugh, and R. Nay, *Promoting a continuation of self and normality: person-centred care as described by people with dementia, their family members and aged care staff.* Journal of clinical nursing, 2010. **19**(17-18): p. 2611-2618.

2. Hutchison, B., et al., *Primary health care in Canada: systems in motion.* Milbank Quarterly, 2011. **89**(2): p. 256-288.

3. Stiggelbout, A.M., et al., *Shared decision making: really putting patients at the centre of healthcare.* Bmj, 2012. **344**(S 28).

4. Wolfs, C.A., et al., *Rational decision-making about treatment and care in dementia: A contradiction in terms?* Patient Education & Counseling, 2012. **87**(1): p. 43-48 6p.

5. Edvardsson, D., B. Winblad, and P.-O. Sandman, *Person-centred care of people with severe Alzheimer's disease: current status and ways forward.* The Lancet Neurology, 2008. **7**(4): p. 362-367.

6. McCormack, B., *Person-centredness in gerontological nursing: an overview of the literature.* Journal of clinical nursing, 2004. **13**(s1): p. 31-38.

For peer review only - http://bmjopen.bmj.com/site/about/guidelines.xhtml

7. CQC, C.Q.C., *Care Homes.* 2009.

8. Bartlett, R. and D. O'Connor, *Broadening the dementia debate: Towards social citizenship*. 2010: Policy Press.

9. Wilkinson, H., *Empowerment and decision-making for people with dementia: the use of legal interventions in Scotland.* Aging Ment Health, 2001. **5**(4): p. 322-8.

10. Bekker, H.L., *Using decision-making theory to inform clinical practice.* Shared decision-making in health care: achieving evidence-based patient choice, 2009. **2**: p. 45-51.

11. Smebye, K.L., Kirkevold, M. & Engedal, K. , *How do persons with dementia participate in decision making related to health and daily care? a multi-case study.* BMC Health Serv Res, 2012. **12**: p. 241.

12. Tyrrell, J.G., N.: Myslinski, M., *Freedom of choice and decision-making in health and social care: views of older patients with early-stage dementia and their carers.* Dementia (14713012), 2006. **5**(4): p. 479-502 24p.

13. Nations, U., *Convention on the Rights of Persons with Disabilities (CRPD)*, U. Nations, Editor. 2006, Division for Social Policy and Development Disability: New York.

14. Manthorpe, J.S., K., *Changing practice: Adapting to the Mental Capacity Act 2005.* Social Care and Neurodisability, 2013. **4**(3): p. 124-133.

15. Affairs, D.o.C., *Mental Capacity Act 2005*, D.o.C. Affairs, Editor. 2005, HMSO London.

16. Affairs, D.o.C., *Mental Capacity Act 2005; code of practice.* . 2007, TSO: London.

17. Boyle, G., *The Mental Capacity Act 2005: promoting the citizenship of people with dementia?* Health & Social Care in the Community, 2008. **16**(5): p. 529-537 9p.

18. Manthorpe, J.S., K., *Mental capacity and dementia: A review. Part 2.* Journal of Dementia Care, 2013. **21**(1): p. 35-38.

19. Goodman, C., et al., *Preferences and priorities for ongoing and end-of-life care: A qualitative study of older people with dementia resident in care homes.* International journal of nursing studies, 2013. **50**(12): p. 1639-1647.

20. Mathie, E., et al., *An uncertain future: the unchanging views of care home residents about living and dying.* Palliative Medicine, 2012. **26**(5): p. 734-743.

For peer review only

For peer review only - http://bmjopen.bmj.com/site/about/guidelines.xhtml

21. Miller, L.M., C.J. Whitlatch, and K.S. Lyons, *Shared decision-making in dementia: A review of patient and family carer involvement.* Dementia, 2014.

22. Larsson, A.T. and J.H. Österholm, *How are decisions on care services for people with dementia made and experienced? A systematic review and qualitative synthesis of recent empirical findings.* International Psychogeriatrics, 2014. **26**(11): p. 1849-1862.

23. Taghizadeh Larsson, A.O., J. H., *How are decisions on care services for people with dementia made and experienced? A systematic review and qualitative synthesis of recent empirical findings.* Int Psychogeriatr, 2014. **26**(11): p. 1849-62.

24. Menne, H.L.W., C. J., *Decision-making involvement of individuals with dementia.* Gerontologist, 2007. **47**(6): p. 810-9.

25. Mariani, E., et al., *Shared decision-making on a 'life-and-care plan'in long-term care facilities: research protocol.* Nursing Open, 2016.

26. Orrell, M., et al., *The needs of people with dementia in care homes: the perspectives of users, staff and family caregivers.* International Psychogeriatrics, 2008. **20**(05): p. 941-951.

27. Daly, R., F. Bunn, and C. Goodman, *Shared decision-making for people living with dementia in extended care settings: protocol for a systematic review.* BMJ open, 2016. **6**(11): p. e012955.

28. Moher, D., et al., *Preferred reporting items for systematic review and meta-analysis protocols (PRISMA-P) 2015 statement.* Syst Rev, 2015. **4**: p. 1.

29. Braun, V. and V. Clarke, *Using thematic analysis in psychology.* Qualitative research in psychology, 2006. **3**(2): p. 77-101.

30. Kitwood, T., *The experience of dementia.* Aging & Mental Health, 1997. **1**(1): p. 13-22.

31. Kitwood, T., *The concept of personhood and its relevance for a new culture of dementia care*. 1997: London: Routledge.

32. Kitwood, T., *Dementia Reconsidered*. 1997, Buckingham, UK: Open University Press.

33. Laidsaar-Powell, R., et al., *Physician–patient–companion communication and decision-making: a systematic review of triadic medical consultations.* Patient Education and Counseling, 2013. **91**(1): p. 3-13.

For peer review only

34. Légaré, F., et al., *Barriers and facilitators to implementing shared decision-making in clinical practice: Update of a systematic review of health professionals' perceptions.* Patient Education and Counseling, 2008. **73**(3): p. 526-535.

35. Kmet L, L.R., Cook L., *Standard Quality Assessment Criteria for Evaluating Primary Research Papers from a Variety of Fields*. 2004, Alberta Heritage Foundation for Medical Research: Edmonton.

36. Noyes J, H.K., Booth A, Harris J, Harden A, Popay J, Pearson A, Cargo M, and Pantoja T, *Qualitative research and Cochrane reviews. In:*, in *Cochrane Handbook for Systematic Reviews of Interventions* G.S. Higgins JPT, Editor. 2015, The Cochrane Collaboration: http://qim.cochrane.org/supplemental-handbook-guidance.

37. Hannes, K., *Critical appraisal of qualitative research*, in *Supplementary Guidance for Inclusion of Qualitative Research in Cochrane Systematic Reviews of Interventions.*, B.A. Noyes J, Hannes K, Harden A, Harris J, Lewin S, Lockwood C. , Editor. 2011, Cochrane Collaboration Qualitative Methods Group: http://cqrmg.cochrane.org/supplemental-handbook-guidance.

38. Clarke, V. and V. Braun, *Thematic analysis*, in *Encyclopedia of critical psychology*. 2014, Springer. p. 1947-1952.

39. Bailey, R.W., P.: Dymond, S., *A visual aid to decision-making for people with intellectual disabilities.* Research in Developmental Disabilities, 2011. **32**(1): p. 37-46 10p.

40. Boyle, G., *Recognising the agency of people with dementia.* Disability & Society, 2014. **29**(7): p. 1130-1144 15p.

41. Clarke, A.M.D., M. F., *Communication and decision making among residents with dementia.* Geriaction, 2004. **22**(3): p. 17-24 8p.

42. Feinberg, L.F. and J. Whitlatch, *Decision-making for persons with cognitive impairment and their family caregivers.* American journal of Alzheimer's disease and other dementias, 2002. **17**(4): p. 237-244.

43. Ferm, U.S., A.: Sundin, L.: Hartelius, L., *Using Talking Mats to support communication in persons with Huntington's Disease.* International Journal of Language & Communication Disorders, 2010. **45**(5): p. 523-536 14p.

44. Fetherstonhaugh, D.T., L.: Nay, R., *Being central to decision making means I am still here!: the essence of decision making for people with dementia.* J Aging Stud, 2013. **27**(2): p. 143-50.

For peer review only - http://bmjopen.bmj.com/site/about/guidelines.xhtml

45. Godwin, B., *Colour consultation with dementia home residents and staff.* Quality in Ageing & Older Adults, 2014. **15**(2): p. 102-119 18p.

46. Hirschman, K.B.J., C. M.: James, B. D.: Xie, S. X.: Karlawish, J. H., *Do Alzheimer's disease patients want to participate in a treatment decision, and would their caregivers let them?* Gerontologist, 2005. **45**(3): p. 381-8.

47. Horton-Deutsch, S.T., Prudence: Evans, Rebecca, *Health care decision-making of persons with dementia.* Dementia, 2007. **6**(1): p. 105-120.

48. Kjellberg, A., *More or less independent.* Disability & Rehabilitation, 2002. **24**(16): p. 828-840 13p.

49. Menne, H.L.T., S. S.: Whitlatch, C. J.: Feinberg, L. F., *Decision-making involvement scale for individuals with dementia and family caregivers.* Am J Alzheimers Dis Other Demen, 2008. **23**(1): p. 23-9.

50. Milte, C.M.R., Julie: Davies, Owen: Whitehead, Craig: Masters, Stacey: Crotty, Maria, *Family meetings for older adults in intermediate care settings: the impact of patient cognitive impairment and other characteristics on shared decision making.* Health Expectations, 2015. **18**(5): p. 1030-1040.

51. Murphy, J.O., T., *The use of Talking Mats to support people with dementia and their carers to make decisions together.* Health Soc Care Community, 2013. **21**(2): p. 171-80.

52. Samsi, K. and J. Manthorpe, *Everyday decision-making in dementia: findings from a longitudinal interview study of people with dementia and family carers.* International Psychogeriatrics, 2013. **25**(6): p. 949-961 13p.

53. Span, M., *Developing an interactive web tool to facilitate shared decision-making in dementia care networks: a participatory journey*. 2016, Amsterdam: Vrije Universiteit.

54. Whitlatch, C.J.F., Lynn Friss Tucke, Shandra S., *Measuring the Values and Preferences for Everyday Care of Persons with Cognitive Impairment and Their Family Caregivers.* The Gerontologist, 2005. **45**(3): p. 370-380.

55. Elwyn, G., et al., *Investing in deliberation: a definition and classification of decision support interventions for people facing difficult health decisions.* Medical Decision Making, 2010. **30**(6): p. 701-711.

56. Thompson, A.G., *The meaning of patient involvement and participation in health care consultations: a taxonomy.* Social science & medicine, 2007. **64**(6): p. 1297-1310.

For peer review only - http://bmjopen.bmj.com/site/about/guidelines.xhtml

57. Groen-van de Ven, L., et al., *Involvement of people with dementia in making decisions about their lives: a qualitative study that appraises shared decision-making concerning daycare.* BMJ open, 2017. **7**(11): p. e018337.

58. Society, A.s., *Turning Up the Volume: unheard voices of people with dementia*. 2017, Alzheimer's Society: https://www.ipsos.com/sites/default/files/2017-05/dementia-alzheimers-society-may-2017.pdf. p. 44.

59. Mariani, E., et al., *Shared decision-making in dementia care planning: barriers and facilitators in two European countries.* Aging & mental health, 2017. **21**(1): p. 31-39.

For peer review only

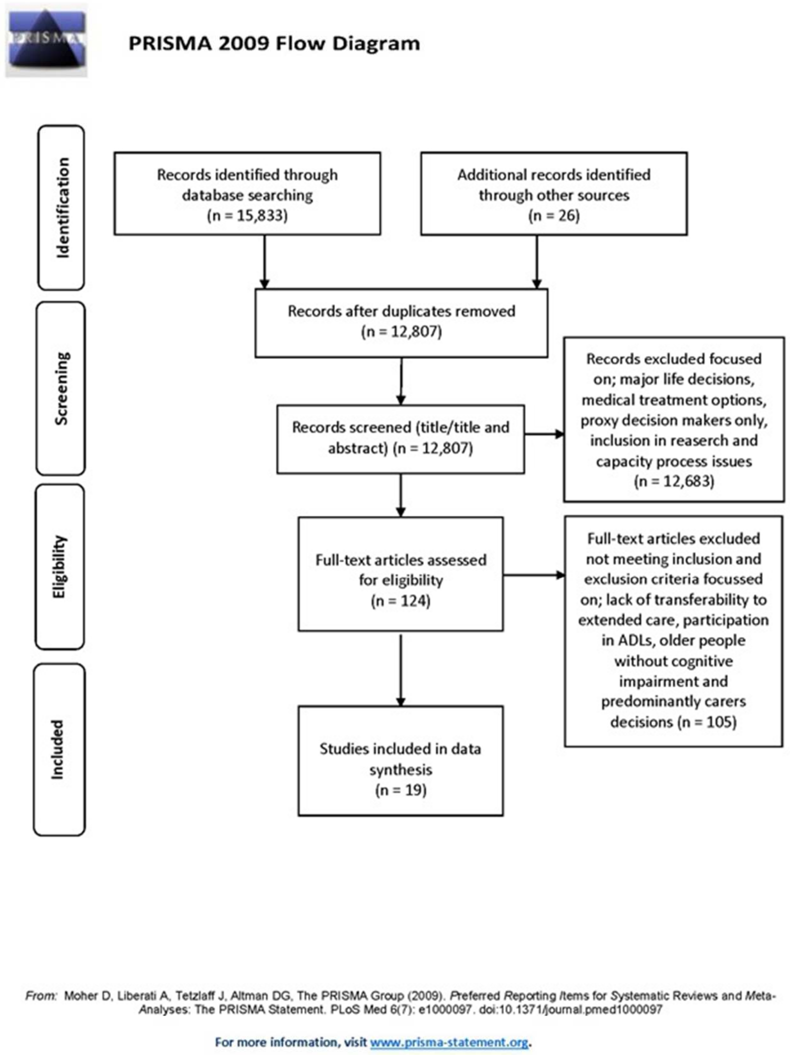

60x78mm (300 x 300 DPI)

PRISMA 2009 Checklist SHARED DECISION-MAKING FOR PEOPLE LIVING WITH DEMENTIA IN EXTENDED CARE SETTINGS: A SYSTEMATIC REVIEW

Section/topic	#	Checklist item	Reported on page #
**TITLE**			
Title	1	Identify the report as a systematic review, meta-analysis, or both.	3,4
**ABSTRACT**			
Structured summary	2	Provide a structured summary including, as applicable: background; objectives; data sources; study eligibility criteria, participants, and interventions; study appraisal and synthesis methods; results; limitations; conclusions and implications of key findings; systematic review registration number.	2
**INTRODUCTION**			
Rationale	3	Describe the rationale for the review in the context of what is already known.	3-4
Objectives	4	Provide an explicit statement of questions being addressed with reference to participants, interventions, comparisons, outcomes, and study design (PICOS).	4-5
**METHODS**			
Protocol and registration	5	Indicate if a review protocol exists, if and where it can be accessed (e.g., Web address), and, if available, provide registration information including registration number.	4
Eligibility criteria	6	Specify study characteristics (e.g., PICOS, length of follow-up) and report characteristics (e.g., years considered, language, publication status) used as criteria for eligibility, giving rationale.	5
Information sources	7	Describe all information sources (e.g., databases with dates of coverage, contact with study authors to identify additional studies) in the search and date last searched.	6-7
Search	8	Present full electronic search strategy for at least one database, including any limits used, such that it could be repeated.	6-7
Study selection	9	State the process for selecting studies (i.e., screening, eligibility, included in systematic review, and, if applicable, included in the meta-analysis).	8
Data collection process	10	Describe method of data extraction from reports (e.g., piloted forms, independently, in duplicate) and any processes for obtaining and confirming data from investigators.	8
Data items	11	List and define all variables for which data were sought (e.g., PICOS, funding sources) and any assumptions and simplifications made.	5
Risk of bias in individual studies	12	Describe methods used for assessing risk of bias of individual studies (including specification of whether this was done at the study or outcome level), and how this information is to be used in any data synthesis.	8
Summary measures	13	State the principal summary measures (e.g., risk ratio, difference in means).	N/A
Synthesis of results	14	Describe the methods of handling data and combining results of studies, if done, including measures of consistency (e.g., I^2) for each meta-analysis.	N/A

For peer review only

For peer review only - http://bmjopen.bmj.com/site/about/guidelines.xhtml

PRISMA 2009 Checklist SHARED DECISION-MAKING FOR PEOPLE LIVING WITH DEMENTIA IN EXTENDED CARE SETTINGS: A SYSTEMATIC REVIEW

Section/topic	#	Checklist item	Reported on page #
Risk of bias across studies	15	Specify any assessment of risk of bias that may affect the cumulative evidence (e.g., publication bias, selective reporting within studies).	8
Additional analyses	16	Describe methods of additional analyses (e.g., sensitivity or subgroup analyses, meta-regression), if done, indicating which were pre-specified.	N/A
**RESULTS**			
Study selection	17	Give numbers of studies screened, assessed for eligibility, and included in the review, with reasons for exclusions at each stage, ideally with a flow diagram.	8-10
Study characteristics	18	For each study, present characteristics for which data were extracted (e.g., study size, PICOS, follow-up period) and provide the citations.	10
Risk of bias within studies	19	Present data on risk of bias of each study and, if available, any outcome level assessment (see item 12).	9
Results of individual studies	20	For all outcomes considered (benefits or harms), present, for each study: (a) simple summary data for each intervention group (b) effect estimates and confidence intervals, ideally with a forest plot.	11-17
Synthesis of results	21	Present results of each meta-analysis done, including confidence intervals and measures of consistency.	N/A
Risk of bias across studies	22	Present results of any assessment of risk of bias across studies (see Item 15).	9
Additional analysis	23	Give results of additional analyses, if done (e.g., sensitivity or subgroup analyses, meta-regression [see Item 16]).	N/A
**DISCUSSION**			
Summary of evidence	24	Summarize the main findings including the strength of evidence for each main outcome; consider their relevance to key groups (e.g., healthcare providers, users, and policy makers).	17-18
Limitations	25	Discuss limitations at study and outcome level (e.g., risk of bias), and at review-level (e.g., incomplete retrieval of identified research, reporting bias).	18
Conclusions	26	Provide a general interpretation of the results in the context of other evidence, and implications for future research.	19
**FUNDING**			
Funding	27	Describe sources of funding for the systematic review and other support (e.g., supply of data); role of funders for the systematic review.	20

From: Moher D, Liberati A, Tetzlaff J, Altman DG, The PRISMA Group (2009). Preferred Reporting Items for Systematic Reviews and Meta-Analyses: The PRISMA Statement. PLoS Med 6(7): e1000097. doi:10.1371/journal.pmed1000097

For more information, visit: www.prisma-statement.org.

For peer review only - http://bmjopen.bmj.com/site/about/guidelines.xhtml
